# Utilizing High X-ray Energy Photon-In Photon-Out Spectroscopies and X-ray Scattering to Experimentally Assess the Emergence of Electronic and Atomic Structure of ZnS Nanorods

**DOI:** 10.1021/jacs.4c10257

**Published:** 2024-11-25

**Authors:** Lars Klemeyer, Tjark L. R. Gröne, Cecilia de Almeida Zito, Olga Vasylieva, Melike Gumus Akcaalan, Sani Y. Harouna-Mayer, Francesco Caddeo, Torben Steenbock, Sarah-Alexandra Hussak, Jagadesh Kopula Kesavan, Ann-Christin Dippel, Xiao Sun, Andrea Köppen, Viktoriia A. Saveleva, Surender Kumar, Gabriel Bester, Pieter Glatzel, Dorota Koziej

**Affiliations:** † Institute for Nanostructure and Solid-State Physics, Center for Hybrid Nanostructures, University of Hamburg, Luruper Chaussee 149, Hamburg 22761, Germany; ‡ The Hamburg Center for Ultrafast Imaging, Hamburg 22761, Germany; § Department of Chemistry, University of Hamburg, HARBOR, Luruper Chaussee 149, Hamburg 22761, Germany; ∥ Deutsches Elektronen-Synchrotron DESY, Notkestraße 85, Hamburg 22607, Germany; ⊥ Department of Chemistry, University of Hamburg, Grindelallee 117, Hamburg 20146, Germany; # ESRF, The European Synchrotron, 71 Avenue des Martyrs, CS40220, Grenoble 38043, France; ∇ Institute of Integrated Natural Science, University of Koblenz, Universitätsstraße 1, Koblenz 56070, Germany

## Abstract

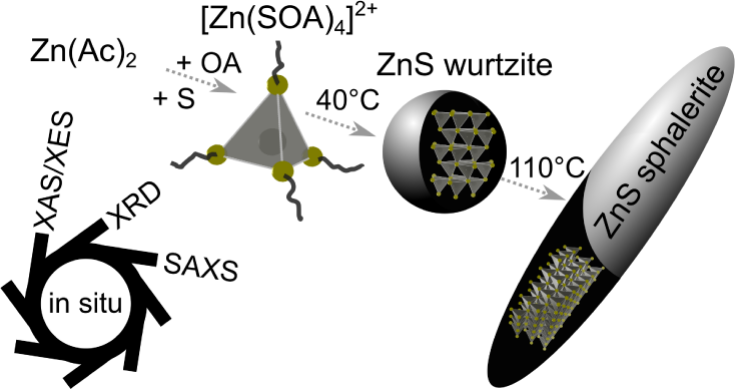

The key to controlling the fabrication process of transition metal sulfide nanocrystals is to understand the reaction mechanism, especially the coordination of ligands and solvents during their synthesis. We utilize *in situ* high-energy resolution fluorescence detected X-ray absorption spectroscopy (HERFD-XAS) as well as *in situ* valence-to-core X-ray emission spectroscopy (vtc-XES) combined with density functional theory (DFT) calculations to identify the formation of a tetrahedral [Zn(OA)_4_]^2+^ and an octahedral [Zn(OA)_6_]^2+^ complex, and the ligand exchange to a tetrahedral [Zn(SOA)_4_]^2+^ complex (OA = oleylamine, OAS = oleylthioamide), during the synthesis of ZnS nanorods in oleylamine. We observe *in situ* the transition of the electronic structure of [Zn(SOA)_4_]^2+^ with a HOMO/LUMO gap of 5.0 eV toward an electronic band gap of 4.3 and 3.8 eV for 1.9 nm large ZnS wurtzite nanospheres and 2 × 7 nm sphalerite nanorods, respectively. Thus, we demonstrate how *in situ* multimodal X-ray spectroscopy and scattering studies can not only resolve structure, size, and shape during the growth and synthesis of NPs in organic solvents and at high temperature but also give direct information about their electronic structure, which is not readily accessible through other techniques.

## Introduction

Zinc sulfide (ZnS) plays an important role in a wide range of applications, such as optoelectronic devices and field emitters,^1^ photodetectors,^2^ photocatalysis,^3−5^ and protective shell material in core–shell nanostructures.^6,7^ Particularly, anisotropic structures like nanorods or nanosheets are of great interest, due to charge carrier confinement in different dimensions.^8^ ZnS exhibits two crystal structures, sphalerite or zinc blende (cubic) and wurtzite (hexagonal) phases.^9^ The phase transition from sphalerite to wurtzite can be either induced by thermal annealing at temperatures above 1000 °C^10−12^ or directly in the solvothermal synthesis of nanocrystals (NC) at temperatures around 150 °C.^13−17^

Among nonaqueous synthesis routes,^17−21^ the combination of elemental sulfur and oleylamine is widely explored in the synthesis of transition metal sulfides,^18^ particularly of ZnS,^19,22^ but the understanding of their reaction with the metal precursor is still under debate.^21^ It has been reported that elemental sulfur and oleylamine form various sulfur species with the formation of H_2_S,^18,23^ but so far, it is not clear which sulfur species interacts with the metal precursor and actively takes part in the formation of transition metal sulfides. The interaction between zinc acetate (Zn(Ac)_2_) and elemental sulfur in oleylamine has not been studied *in situ*.

Optical methods like visible light fluorescence and absorption, commonly used for studying the semiconducting quantum dots, can hardly be utilized to follow *in situ* changes of the electronic structure of Zn species in solution due to the large ZnS band gap that overlaps with the absorption of the organic ligands in the reaction solution.^24^ X-ray absorption spectroscopy (XAS),^25,26^ particularly high-energy resolution fluorescence detected X-ray absorption spectroscopy (HERFD-XAS), is element-specific and offers sensitivity to the local environment around the absorbing atom.,^27−30^ Data acquisition for HERFD-XAS is challenging due to the need to balance data quality, time resolution, and X-ray radiation damage. Additionally, valence-to-core X-ray emission spectroscopy (vtc-XES) enables to probe the valence orbitals and can provide detailed information e.g., about ligand bonds to a metal center by mapping the occupied molecular orbitals.^31−33^ The vtc-XES signal at the Zn edge is over 100 times weaker than core-to-core XES transitions, necessitating extended data acquisition times.^34−36^

Combining HERFD-XAS with X-ray scattering provides a comprehensive overview of the reaction pathways across various length scales,^36−38^ by revealing a wide range of structural, chemical, and electronic properties of materials.^39−44^ However, there are no combined *in situ* HERFD-XAS/vtc-XES studies on the nucleation and growth of nanoparticles at high temperatures in solution at relevant time scales.

In this work, we conduct *in situ* experiments on a fourth-generation synchrotron, which provides enhanced flux and thus enables faster acquisition of vtc-XES data. We present a methodology to elucidate the chemical pathways leading to the formation of ZnS NC in the oleylamine-sulfur system, together with the emergence of their electronic properties by complementary *in situ* HERFD-XAS, vtc-XES, PXRD, and SAXS. Thereby, we propose the formation of [Zn(OA)_4_]^2+^, [Zn(OA)_6_]^2+^ and [Zn(SOA)_4_]^2+^ complexes (OA = oleylamine, SOA = oleylthioamide) and track their reaction to ZnS NC in the wurtzite phase (w-ZnS) and the transition to ZnS nanorods in the sphalerite phase (s-ZnS).

## Results and Discussion

The dissolution of Zn(Ac)_2_ and elemental sulfur in oleylamine leads to the formation of a tetrahedral [Zn(SOA)_4_]^2+^ complex, in which Zn^2+^ is coordinated by four thioamide ligands. When this complex is heated to 155 °C, ZnS nanoparticles in the wurtzite phase (w-ZnS) form as an intermediate, which partially converts to ZnS nanorods in the sphalerite phase (s-ZnS). The findings of this work are arranged in three sections. First, we present the characterization of mixing precursors at room temperature. Then, we provide *in situ* characterization of the electronic structures during the synthesis of ZnS. Finally, we monitor the *in situ* nucleation, phase transition, and growth of ZnS.

## Chemical Transformation of Zn(Ac)_2_ and Elemental Sulfur in Oleylamine

**Figure 1:**
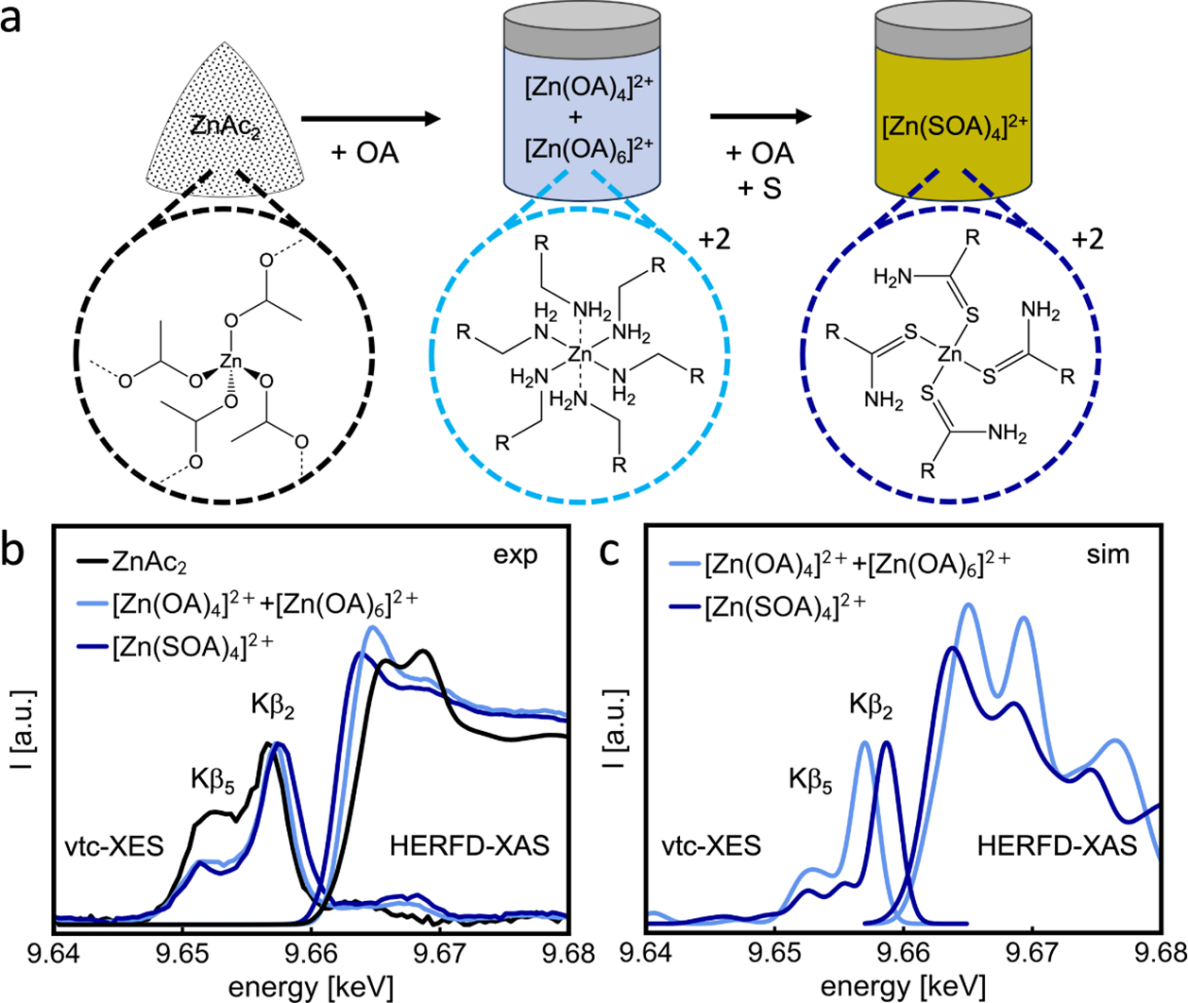
Identifying Zn coordination by HERFD-XAS analysis. (a) Schematics of the reaction pathway from Zn(Ac)_2_ precursor (black dashed circle) to a tetrahedral [Zn(SOA)_4_]^2+^ complex, (dark blue), which undergoes an intermediate step: a mixture of tetrahedral [Zn(OA)_4_]^2+^ (60%) and octahedral [Zn(OA)_6_]^2+^ (40%) complexes (light blue). The four and six oleylamine chains in the mixed complexes are replaced by four of their corresponding thioamide derivatives upon the addition of sulfur to the mixture. (b) Comparison of experimental HERFD-XAS and vtc-XES spectra of Zn(Ac)_2_ (black line), Zn(Ac)_2_ dissolved in oleylamine (light blue solid line) forming a mixture of [Zn(OA)_4_]^2+^ and [Zn(OA)_6_]^2+^, and Zn(Ac)_2_ dissolved in oleylamine with sulfur (dark blue solid line), resulting in the [Zn(SOA)_4_]^2+^ complex. (c) Theoretical XAS and vtc-XES spectra obtained using DFT calculations of the proposed tetrahedral [Zn(OA)_4_]^2+^ mixed with octahedral [Zn(OA)_6_]^2+^ complexes at the ratio 60:40, and the [Zn(SOA)_4_]^2+^ complex. Peaks in vtc-XES spectra above the Fermi level are described by multielectron excitations in the literature.^46,47^ (*R* = (CH_2_)_7_(HC = CH)(CH_2_)_7_(CH_3_)).

We first investigate the coordination of Zn^2+^ ions at room temperature after the dissolution of Zn(Ac)_2_ in oleylamine before and after the addition of elemental sulfur. To unveil the nature of the Zn complexes formed, we compared the HERFD-XAS and vtc-XES measurements with the theoretical spectra simulated by density functional theory (DFT) using the ORCA code, as shown in Figure 1. The spectrum of Zn(Ac)_2_ dissolved in oleylamine (light blue) exhibits a 0.8 eV shift in the XAS E_0_ position (9.665 keV) to lower energies and a 0.6 eV shift of the Kβ_2,5_ peak (9.657 keV) to higher energies, compared to the Zn(Ac)_2_ reference (black), while the intensity of the white line increased. The white line corresponds to the position of maximal intensity in XAS, while E_0_ reflects the maximum in the first derivative. The energy shifts might suggest the replacement of the Zn–O coordination by Zn–N coordination while the increased white line intensity indicates a change in the coordination of the Zn atom, from tetrahedral to partly octahedral. By comparing the experimental and simulated spectra, we propose that the dissolution of Zn(Ac)_2_ in oleylamine leads to the displacement of the acetate ligands by four and six oleylamine molecules, resulting in a mixture of a tetrahedral [Zn(OA)_4_]^2+^(60%) and an octahedral [Zn(OA)_6_]^2+^ (40%) complex, visualized in Figure 1b. The DFT calculations show that the mixture of tetrahedral and octahedral N-coordinated complexes matches best to the experimental data as shown in Figures 1b,SI1a, and SI2.

The addition of elemental sulfur (dark blue) leads to a 0.6 eV shift of the E_0_ to lower energies, while the Kβ_2,5_ peak shifts 0.6 eV to higher energies, compared to the mixture without sulfur, as shown in Figure 1c. Furthermore, the addition of sulfur also results in a decrease in the white line intensity. The energy shifts result from a ligand exchange around Zn, where the Zn–N coordination is replaced by Zn–S. The decrease in white line intensity indicates that the coordination number of Zn decreased from a mixture of four and six to only four. The theoretical XAS and XES spectra obtained from DFT (blue dashed line) simulations reveal that four and six oleylamine chains of the initial [Zn(OA)_4_]^2+^ and [Zn(OA)_6_]^2+^ complexes are replaced by four of their corresponding thioamide derivatives, resulting in the [Zn(SOA)_4_]^2+^ complex shown in Figure 1a. The formation of other Zn–S coordination, like [Zn(H_2_S)_4_]^2+^ is ruled out in Figure SI1b. The calculations of [Zn(OA)_4_]^2+^, [Zn(OA)_6_]^2+^, and [Zn(SOA)_4_]^2+^ were compared with a simulation of a molecular unit of Zn(Ac)_2_ in Figure SI3.^45^ A detailed analysis of the mixing fractions between [Zn(OA)_4_]^2+^ and [Zn(OA)_6_]^2+^ is shown in Figure SI4. The transitions in vtc-XES, as well as the donor orbital for each ORCA calculation, are shown in Figure SI5.

The change from a partly octahedral to a tetrahedral coordination geometry might be related to the steric hindrance of sulfur atoms, which occupy more space compared to nitrogen. Additional information on the formation of the tetrahedral [Zn(SOA)_4_]^2+^ complex is reported in Figure SI6. To match the absolute energy scale of the theoretical and experimental spectra, the calculated energy positions were corrected, as described in Supporting Information.

Thus, our experiments demonstrate that oleylamine and sulfur react already at room temperature in the presence of Zn^2+^ ions, forming the thioamide derivative of oleylamine, which so far has been evidenced by NMR and SAXS only at elevated reaction temperatures.^13,18,23^^13^C NMR spectroscopy shows evidence of the thioamide functional group only in mixtures of oleylamine and sulfur heated at temperatures above 170 °C (see Figures SI7,8 and Supplementary Notes).

## *In Situ* HERFD-XAS and vtc-XES Resolving the Electronic Structures during the Synthesis

**Figure 2:**
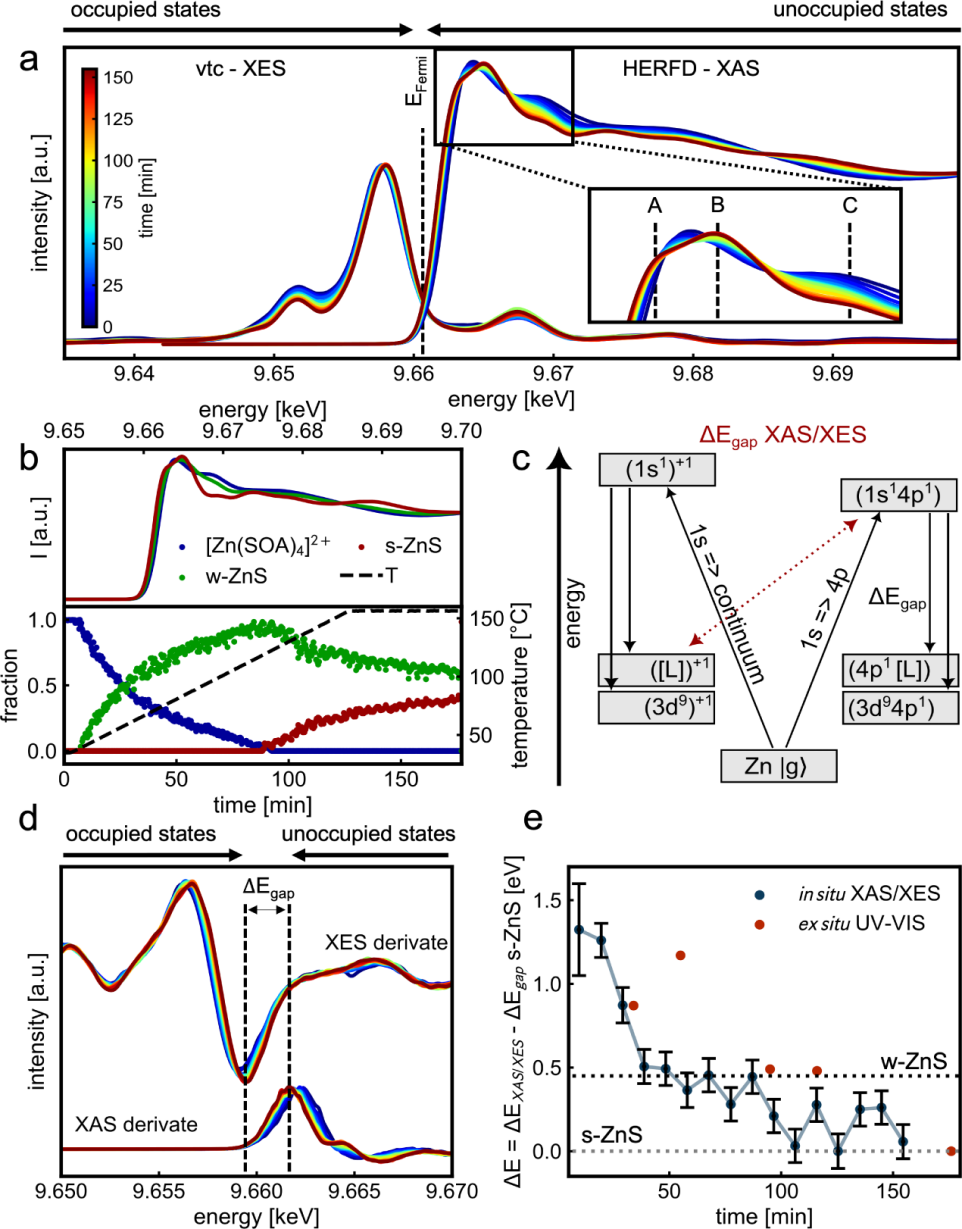
*In situ* X-ray spectroscopy of the synthesis of s-ZnS. (a) *In situ* Zn K-edge vtc-XES and HERFD-XAS spectra of the synthesis of s-ZnS where changes in the XAS are highlighted in the inlet. (b) MCR-ALS analysis of *in situ* HERFD-XAS data reveals individual contributions of three independent compounds, the [Zn(SOA)_4_]^2+^ complex, w-ZnS, and s-ZnS. (c) Schematic interstate transitions during nonresonant (left) and resonant (right) excitation result in a difference in the calculated band gap. (d) The difference between the minimum of the derivation of the XES signal and the maximum of the derivation of the XAS signal reflects the HOMO/LUMO gap during the preparation of s-ZnS. (e) The HOMO/LUMO gap values determined by *in situ* XAS/XES during the reaction in solution are compared with the HOMO/LUMO optical gap values determined with *ex situ* UV–vis analysis of unwashed aliquots (red).

In Figure 2a, we present the *in situ* vtc-XES and HERFD-XAS data sets, which track the reaction of [Zn(SOA)_4_]^2+^ to s-ZnS NC. The HERFD-XAS data reveal a splitting of the white line during the formation of s-ZnS, as highlighted in the inset with peaks A and B. While the evolving shoulder (A) shifts to lower energy, which is typical for ZnS, the absorption maximum (B) shifts to higher energies.^29,48^ Notably, the intensity of the white line remains almost constant during the reaction, which indicates a constant tetrahedral coordination of the Zn atoms through the reaction. The broadening of peaks B and C results in a nonglobal minima between the peaks which is untypical for s-ZnS and might suggest the coformation of w-ZnS. To analyze the fraction of w-ZnS in the final product, multivariate curve resolution-alternating least-squares (MCR-ALS) analysis, a multicomponent analysis, was performed.^49,50^ Further information about the MCR-ALS method is available in the SI, Figures SI9,10 and Tables SI1–2.

The MCR-ALS analysis allows us to track the time- and temperature-dependent concentration profile of all components in the reaction, as shown in Figure 2b. In total, three different components are present during the reaction. In addition to the [Zn(SOA)_4_]^2+^ starting complex and the s-ZnS, w-ZnS is identified as a byproduct of the reaction. The recovered spectra of these components are shown in Figure 2b **top**, while their concentration profile is shown in Figure 2b **bottom**. At 40 °C (15 min), the [Zn(SOA)_4_]^2+^ complex begins to convert to w-ZnS. Upon reaching 110 °C (85 min), the transformation of the [Zn(SOA)_4_]^2+^ complex into w-ZnS is completed, marking the onset of the formation of s-ZnS. As the reaction temperature reaches 155 °C (130 min), the formation of s-ZnS persists, resulting in a final product composition of 60% w-ZnS and 40% s-ZnS.

Beyond the component analysis with MCR-ALS analysis, we combined *in situ* HERFD-XAS data with *in situ* vtc-XES data to monitor the HOMO/LUMO gap during the reaction. For this, we calculated the energy difference between the resonant excitation in HERFD-XAS (1s = > 4p—LUMO) and the highest energetic recombination in vtc-XES (3p = > 1s—HOMO). The total energy scheme for photon-in (Ω) and photon-out (ω) spectroscopy with ground, intermediate, and final states at the Zn edge is shown in Figure 2c. The HOMO/LUMO gap was calculated by the difference between the global minima of the first derivative of the vtc-XES and the global maxima of the first derivative of the HERFD-XAS, as described in Figure 2d. More detailed information regarding the *in situ* HOMO/LUMO gap determination is available in the Supporting Information.

We observe a decrease in the HOMO/LUMO gap (Δ*E*) as the reaction progresses, and the transformation of the [Zn(SOA)_4_]^2+^ complex to the w-ZnS and s-ZnS takes place (Figure 2e). To calibrate the energy scale, we set the E at the end of the reaction to the band gap of s-ZnS (3.8 eV). We determine the HOMO/LUMO gap of the [Zn(SOA)_4_]^2+^ complex to be 5.0 eV. At 155 °C reaction temperature and a ramping rate of 1 °C/min, the HOMO/LUMO gap changes stepwise. First, Δ*E* quickly drops by 0.7 eV from 5.0 to 4.3 eV, which reflects the band gap of w-ZnS during our synthesis. Therefore, we assume that the band structure has already evolved after 40 min. The band gap is comparably high for w-ZnS, which suggests a very small crystallite size due to quantum confinement effects.^51^ To estimate the size of the w-ZnS we have performed atomic effective pseudopotential calculations and obtained, for an electronic gap of 4.3 eV, a diameter of around 2.1 nm, as described in Figure SI11.^52−59^ After 100 min, the value reached 3.8 eV, which coincides with the onset of s-ZnS formation shown in Figure 2b.

Moreover, the band gap Δ*E* estimation is consistent with the values determined by using *ex situ* UV–vis of unwashed aliquots (Figures 2e, SI12 and Table SI). UV–vis analysis struggles to discriminate between organic background and emerging w-ZnS, which explains the discrepancy, especially in the early reaction state.

## Simultaneous *In Situ* SAXS and PXRD Resolving the Atomic Structures during the Synthesis

**Figure 3:**
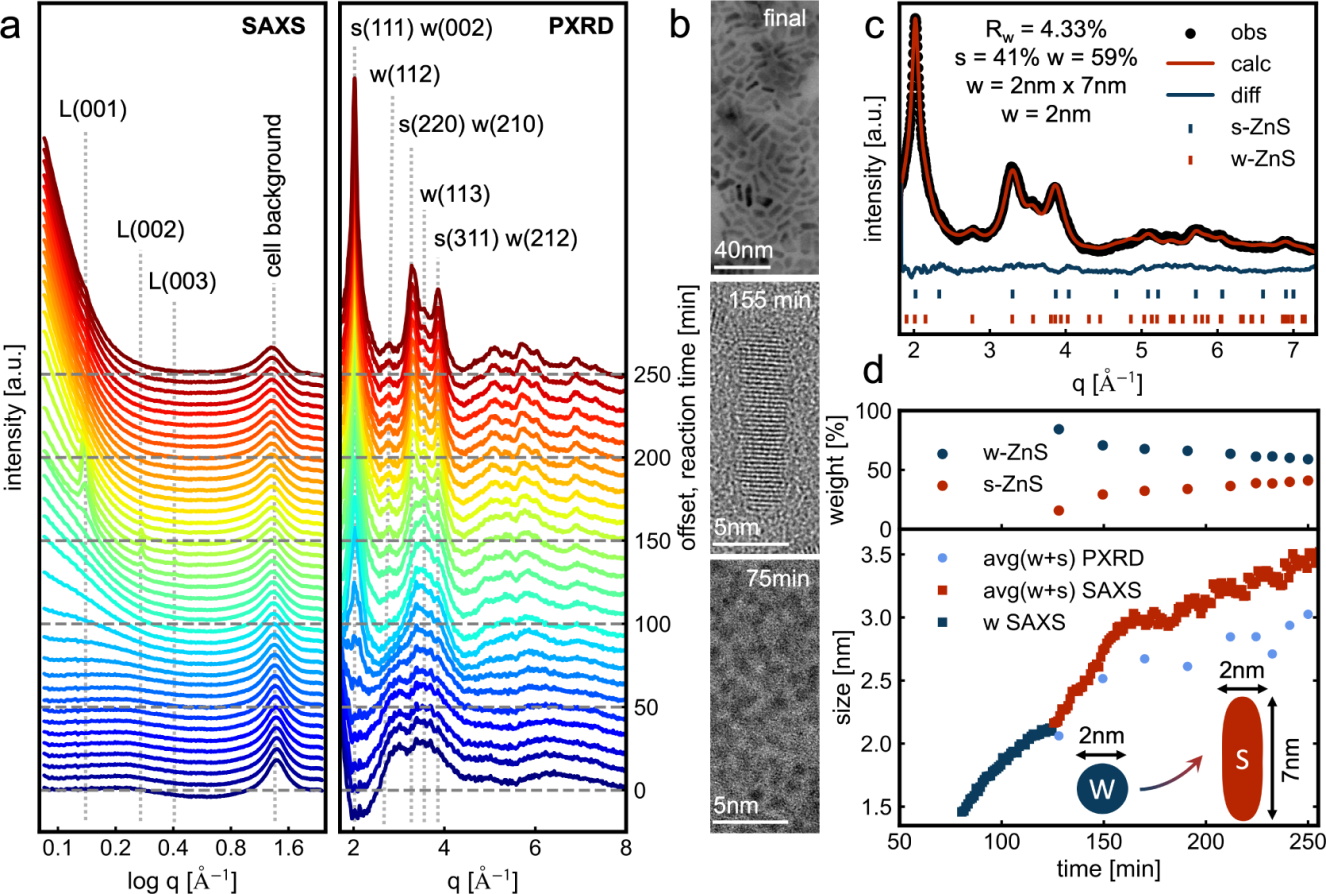
Simultaneous *in situ* SAXS and PXRD, as well as *ex situ* HRTEM analysis.

To characterize the structural evolution of w-ZnS and s-ZnS, *in situ* small-angle X-ray scattering (SAXS) and powder X-ray diffraction (PXRD) was performed and compared with *ex situ* high-resolution transmission electron microscopy (HRTEM) images. The *in situ* SAXS data (Figure 3a) show an increased intensity at low q starting at approximately 70 min, indicating particle formation during the reaction. The particle size was calculated by applying a spherical fitting model to the experimental data with the results shown in Figure 3d. The energy of >100 keV and the resulting high q-min of around 0.1 Å^–1^ restricted the resolution to structures larger than 8 nm. However, the high background (lamellar, solvent) prevented us from deconvoluting the data into distinct spherical fits for w-ZnS and rod-like fits for s-ZnS. Further details about the SAXS fitting and background subtraction are available in Figures SI13–16. Furthermore, the formation and dissolution of oleylamine lamellar structures were observed in the presence of zinc ions in solution, as depicted in Figure SI14, aligning with findings from related studies on synthesis under similar reaction conditions.^23^

*In situ* PXRD data corroborate the previously described transformation of w-ZnS to s-ZnS during the reaction. This trend is evident by comparing the intensity ratios between the reflections of w (113) and s(220)/w(210), as depicted in Figure 3a. At around 110 min, the intensities of both reflections are equal. Thereafter, the intensity ratio shifts toward s(220)/w(210) until the reaction concludes. Additionally, PXRD data reveal a strongly preferred growth direction along the s(111) axis. The formation of spherical w-ZnS as an intermediate in the formation of s-ZnS nanorods is already observed in comparable systems,^15^ where the preferred growth direction is explained by an oriented attachment of w-ZnS.^14^

This preferred growth direction in s-ZnS is also evidenced by the HRTEM analysis of aliquots taken throughout the reaction, as shown in Figure 3b. At 75 min, the formation of w-ZnS results in spherical NC with an estimated size of 1.9 ± 0.2 nm, which is in agreement with 2.1 nm obtained by HERFD-XAS/vtc-XES. Detailed size analysis and full images are provided in Figures SI17–19.

While MCR-ALS analysis proposes that the formation of w-ZnS starts at 40 °C, SAXS and PXRD and HRTEM analyses confirm only the formation above 90 °C. This mismatch can be explained by the varying sensitivity of all methods. X-ray spectroscopy can detect already noncrystalline species and even molecular complexes, while SAXS and PXRD are highly sensitive to the crystal structure, size, and shape of ZnS NC.

The preferred growth direction, as well as the fractions of w-ZnS and s-ZnS, can be extracted from the PXRD data through sequential Rietveld refinement, as illustrated for the final PXRD pattern in Figure 3c. The Rietveld analysis fits a spherical model to the w-ZnS with a domain size of approximately 2 nm, while the s-ZnS demonstrates a preferred growth direction along s (111) and a domain 2 nm x 7 nm. Additional information on the sequential Rietveld refinement is given in Supporting Information. The fraction and domain sizes of s-ZnS and w-ZnS change throughout the reaction, as shown in Figures 3d and SI20. To compare the domain sizes calculated from the PXRD data with those obtained from the SAXS fit, the PXRD sizes were averaged, considering the varying phase fractions at different time points. The PXRD sizes are systematically underestimated compared to the SAXS sizes, as they reflect the domain size, whereas SAXS fitting represents the solvation size of nanoparticles in the solution. The ratio between s-ZnS and w-ZnS can be changed by increasing the reaction temperature to 170 °C and the ramping rate to 10 °C/min, as discussed in Figure SI21.

**(a left)***In situ* SAXS analysis shows an increasing intensity at low q, starting after approximately 70 min of reaction, indicating the formation of spherical nanoparticles. The oleylamine lamellae (L) and cell background (≈ 1.5 Å^–1^) were indicated at the top. **(a right)***In situ* PXRD analysis shows the formation of w-ZnS reflections after 70 min, with changes in the relative intensity of the w(210)/s(220) and w(113) peaks over time, revealing the formation of the s-ZnS phase during the reaction. The s(111) peak has comparable high intensity, thus implying a preferred growth direction in the s-ZnS along the s(111) facet. (b) HRTEM analysis of washed aliquots of the reaction reveals the formation of ZnS nanorods after 120 min, which explains the preferred growth direction along s(111), while at 75 min only spherical particles with 1.9 ± 0.2 nm are observed. (c) Rietveld analysis of the final product shows the presence of 41% s-ZnS and 59% w-ZnS. Moreover, the Rietveld analysis refines a spherical domain size of 2 nm in the w-ZnS and anisotropic domain sizes of 2 nm x 7 nm in the s-ZnS. The fraction of s-ZnS to w-ZnS evolves throughout the reaction, as shown in the top panel of (d). The size calculated by fitting the SAXS data with a spherical model is compared to the average size obtained from the Rietveld analysis in the bottom panel of (d). The smallest size we could resolve with Rietveld refinement of PXRD measurements of w-ZnS nanoparticles was 1.7 nm, while the smallest size we could fit within SAXS analysis was around 1.5 nm.

## Conclusion

This work extends the application of Zn K-edge XAS and vtc-XES in inorganic and bioinorganic chemistry to investigate the emergence and growth of nanomaterials in solution at high temperatures. The integration of HERFD-XAS, Kβ_2,5_ XES, and DFT allows for the identification of successive Zn–O, Zn–N, and Zn–S ligand exchanges.

Our findings reveal that even at room temperature, Zn(Ac)_2_ undergoes a reaction with oleylamine to form a mixture of tetrahedral [Zn(OA)_4_]^2+^ and octahedral [Zn(OA)_6_]^2+^ complexes, which, upon sulfur addition, transforms into a tetrahedral [Zn(SOA)_4_]^2+^ complex. This rules out the formation of, e.g., hydrogen sulfide Zn complexes, such as [Zn(H_2_S)_4_]^2+^. By tracking the *in situ* heating of the [Zn(SOA)_4_]^2+^ complex above 155 °C, we observe the successive nucleation and growth of sphalerite and wurtzite ZnS nanostructures. Interestingly, we also monitor the evolution of the HOMO/LUMO gap from 5.0 to 4.3 eV and eventually to 3.8 eV, consistent with the [Zn(SOA)_4_]^2+^ complex and the 2.1 nm wurtzite and sphalerite structures, respectively.

The structural transformation of ZnS was further analyzed through simultaneous *in situ* SAXS and PXRD measurements. Our observations capture the formation of wurtzite spherical ZnS particles, progressing to the transformation into sphalerite ZnS rods oriented predominantly along the (111) axis. The shape, size, and band gap energy of the nanoparticles were validated through *ex-situ* HRTEM and UV–vis spectroscopy of the powder samples.

In conclusion, this study provides detailed insight into the coordination chemistry and structural changes during the synthesis of ZnS nanocrystals. The methodology showcases its potential to monitor structural and electronic transitions during particle growth at elevated temperatures, particularly in scenarios where optical spectroscopy is not feasible. This approach holds promise for the study of other materials with high energy band gaps or in reaction environments where optical spectroscopy is limited and elemental specificity is crucial for analysis in the future.

## Experimental Section

### Chemicals

Zinc(II) acetate (Zn(Ac)_2_) (99.99%, anhydrous), sulfur (99.998% trace metal basis), and oleylamine (≥98% primary amine) were purchased from Sigma-Aldrich. All chemicals were used as received without further purification and stored, except for sulfur, under an argon atmosphere.

### Synthesis of ZnS

The ZnS synthesis was performed in the *in situ* cell adopted from previous work,^36,63,64^ as described in Figure SI22. Two individual solutions were prepared in oleylamine. First, 0.0489 g of Zn(Ac)_2_ powder was dissolved in 1 mL of oleylamine. Then, 0.0128 g of sulfur was dissolved in 2 mL of oleylamine. Both solutions were stirred for one hour at room temperature under an argon atmosphere. The solutions were added into the microreactor in a volume ratio of 2:1 elemental sulfur:Zn(Ac)_2_ solutions in a total volume of 66 and 174 μL for the scattering and spectroscopic experiments, respectively. The reactor was sealed under argon (99.9999%) and heated at a heating rate of 1 °C/min to 155 °C or at a heating rate of 10 °C/min to 170 °C. The *ex situ* samples for UV–vis analysis were prepared under the same conditions in the *in situ* spectroscopy cell.

UV–vis: UV–visible spectra were collected by using the Cary 60 UV–vis spectrometer (Agilent Technologies Inc., US) and a quartz cuvette. The samples were diluted with cyclohexane in the cuvette. The HOMO/LUMO gap and the band gap were calculated using the Tauc-Plot.^65^

NMR: Nuclear magnetic resonance (NMR) spectra were recorded on a Bruker Avance NEO 600 MHz NMR spectrometer using TopSpin 4.1.3 (BRUKER BIOSPIN GmbH, Rheinstetten, Germany) equipped with a 5 mm TCI Cryoprobe cooled with liquid nitrogen, operating at 600.25 MHz and 298.0 K. All chemical shifts were referenced to residual solvent peaks [CDCl_3_: 7.26 ppm (^1^H), 77.2 ppm (^13^C); C_6_D_6_: 7.3 ppm (^1^H), 128.0 ppm (^13^C)]. One-dimensional (1D) ^1^H and two-dimensional (2D) (^1^H,^13^C)-HSQC and 2D (^1^H,^13^C)-HMBC spectra were acquired using standard pulse sequences from the Bruker library. For the 1D ^13^C{^1^H}-NMR spectra (zgpg30) of the reaction mixture at room temperature, 1024 and 10240 scans (NS) were recorded.

### Sample Preparation for NMR Analysis

Sulfur in oleylamine: Under ambient conditions, sulfur powder (9.1 mmol) was introduced in a glass vial with the further addition of oleylamine (45.5 mmol). The solution was stirred at room temperature until all sulfur was dissolved. The reaction mixture was heated to 170 °C using an oil bath with stirring, and aliquots were taken at 60, 100, and 140 °C. When the solution reached 170 °C, the temperature was maintained for 20 min, and the corresponding aliquot was taken. The mixture was further heated to 190 °C and was kept at 200 °C for 40 min before collection of the 200 °C aliquot.

Zn(Ac)_2_ and S in oleylamine: Under ambient conditions, Zn(Ac)_2_ (6.65 mmol) was introduced into a glass vial with further addition of oleylamine (45.5 mmol). The solution was stirred at room temperature for 30 min and sulfur (9.1 mmol) was added to the mixture as one portion followed by a further 50 min of stirring.

STEM and HRTEM analysis: STEM images were taken and probe-corrected with a Regulus 8220 (Hitachi High Technologies Corp., Japan) at an acceleration voltage of 30 kV and by using the BFSTEM acquisition mode. HRTEM images were taken with a JEOL JEM-2200FS (JEOL Ltd., Japan) using an acceleration voltage of 200 kV.

### Beamline Setup and Data Acquisition

The *in situ* HERFD-XAS and vtc-XES spectra were recorded at the ID26 beamline at the European Synchrotron Radiation Facility (ESRF), Grenoble, France. The HERFD-XAS images were collected by measuring the intensity of the Zn Kα main line using a Si(642) crystal in Rowland Geometry while scanning the incident energy. The position of the X-ray beam was moved on the reaction cell to minimize radiation damage. XAS spectra were acquired every 16 s with an energy range from 9.64 to 9.8 keV and a stepsize of 0.2 eV. The vtc-XES spectra were recorded using four Ge(555) crystals in Rowland geometry over a total energy range from 9.63 to 9.71 keV. To decrease the acquisition time, the spectra region from 9.63 to 9.69 keV was measured with energy steps of 0.4 eV and an acquisition time of 532 s (with motor movements), while the range from 9.69 to 9.71 keV was recorded in steps of 2 eV with a total acquisition time of 44 s. To exclude the occurrence of beam damage during the measurements, a beam damage study prior the experiments were performed, as described in Figure SI23.

The X-ray total scattering and SAXS *in situ* data were collected in a SAXS/WAXS combined setup at the second experimental hutch (EH2) at beamline P07 of PETRA III at Deutsches Elektronen-Synchrotron (DESY), Hamburg, Germany.^66^ The total scattering and SAXS data were collected every 0.5 s using two flat panel detectors (Varex XRD4343CT, Varex Imaging Corp., USA) with 2880 × 2880 pixels of 150 × 150 μm^2^ size. During the experiments to synthesize W and S ZnS at 155 °C, the sample-to-detector distances (SDD) were 0.812 m for total scattering and 4.636 m for the SAXS data, determined from the calibration with the LaB_6_ calibrant, at an X-ray beam energy of 103.56 keV. For the synthesis at 170 °C with a 10 °C/min heating rate, the SDD was determined as 0.765 m for total scattering data collection, obtained from calibration with LaB_6_, and 4.613 m for the SAXS, from the CeO_2_ calibrant, at an X-ray beam energy of 103.60 keV.

The *ex situ* total scattering data were taken at Beamline P21.1 at PETRA III, DESY.^S2^ The total scattering data were recorded with a Varex flat panel detector model XRD4343CT at an SDD of 0.377 m. Samples were enclosed in a quartz capillary, and the calibration was carried out by measuring the LaB_6_ calibrant at an X-ray beam energy of 101.60 keV.

The *ex situ* SAXS data shown in Figure SI15 are collected at Beamline P62 at PETRA III, DESY.^67^ The energy was set to 12 keV using a Si(111) monochromator. The beam size was 0.5 × 0.5 mm^2^. The samples were mounted vertically in a multicapillary holder. The sample-to-detector distances were calibrated to be 2.849 m using AgBH. The SAXS signals were collected by an Eiger2 9 M detector.

### Data Processing

The HERFD-XAS and vtc-XES data were processed using a self-written Python code. The vtc-XES data were normalized by the maximum intensity, since an area normalization led to unphysical intensity fluctuations in the *in situ* data set, as described in Figure SI24. FEFF calculations of vtc-XES of w-ZnS and s-ZnS are shown Figure SI25.^68,69^ The determination of the XAS edge position and normalization of the edge jump were performed by using the LARCH-XAFS module.^70^ The spectroscopic data were treated with a Savitzky-Golay filter and further processed with the NumPy and SciPy package.^71,72^ The processed data are compared with raw data in Figure SI26. The simulations of the XAS spectra were carried out using the ORCA 5.0.4 code,^73^ where the initial zinc complexes for DFT optimization were built using Avogadro: an open-source molecular builder and visualization tool. Version 1.2.0.^74^ The Orca input files were adapted from *Stepanic et.al.*(25)

Azimuthal integration of the 2D detector patterns for PXRD and SAXS data was performed with the Python module pyFAI after masking out beam stop shadows, glitches, pixel defects, and noisy pixels.^75^ For the background subtraction, *in situ* total scattering data of sulfur dissolved in oleylamine and pure oleylamine were collected under the same reaction conditions of the ZnS syntheses at 155 and 170 °C, respectively. The background was subtracted from that of the original data set. The data were averaged over 60 frames, corresponding to a 30 s time resolution. The Rietveld refinement was performed with GSAS-II package,^76^ employing a two-phase refinement with ZnS sphalerite and wurtzite phases sharing one particle size parameter. The sphalerite phase (ICSD-230703) and wurtzite phase (ICSD-67453) were taken from the ICSD database. The refinement was carried out in a sequential way, starting from the XRD at the end of the reaction and going backward to earlier reaction times, ensuring a better reliability of the fit.

The fitting of the SAXS data was carried out over the range of 0.08 to 1.8 Å^–1^. An empty capillary background was measured at room temperature and subtracted from the original *in situ* SAXS data set. The SAXS data were averaged over 120 frames (1 min resolution) and 10 frames (5 s resolution) for the ZnS reactions at 155 and 170 °C, respectively. The fit was carried out in SASview 5.0.6^77^ with the DREAM algorithm.^78^ As a fitting function, a plugin was used, which contained a power law, a sphere, a symmetric pseudo-Voigt profile, and a fitting function for the lamellae. The lamellae were fitted with a triplet of asymmetric pseudo-Voigt profiles sharing the ratio of Gauss to Lorentz factor eta and the fwhm. The peak height ratio was kept constant, while q was allowed a relaxation of ±5% of the multiple of the first peak. For the reaction temperature of 170 °C, an additional sphere model was used with a constant radius of 5.11 Å, and to match the background, the first peak of the lamellae was constrained to be a Lorentz profile only, while the second and third peaks shared the ratio of Gauss to Lorentz factor eta.

To improve the grammar and wording in parts of the manuscript and Supporting Information, ChatGPT 4 omni was used for proofreading, following the guidelines for using AI in scientific publications.^79^

## Supporting Information Available

The supporting information contains DFT calculations, NMR analysis, MCR-ALS analysis, band gap analysis, UV–vis analysis, details on the SAXS background processing, PXRD analysis, STEM and HRTEM analysis, information about the reaction cell, and details on the HERFD-XAS and vtc-XES processing. Further, an HERFD-XAS, vtc-XES, PXRD and SAXS study on the reaction carried out at 170 °C reaction temperature with 10 °C/min ramping rate is available (PDF)
