# Supplementary material for: Utilizing High X-ray Energy Photon-In Photon-Out Spectroscopies and X-ray Scattering to Experimentally Assess the Emergence of Electronic and Atomic Structure of ZnS Nanorods
Source: J Am Chem Soc. 2024 Nov 25;146(49):33475–84. doi: 10.1021/jacs.4c10257 (PMC11638900; doi:10.1021/jacs.4c10257)
Supplement: Supplementary file 1 — ja4c10257_si_001.pdf [file ja4c10257_si_001.pdf]

Supporting Information:

Utilizing high X-ray energy photon-in photon-out spectroscopies and X-ray scattering to experimentally assess the emergence of electronic- and atomic structure of ZnS nanorods

Lars Klemeyer,[1] Tjark L. R. Gröne,[1] Cecilia de Almeida Zito,[1] Olga Vasylieva,[1] Melike Gumus Akcaalan,[1] Sani Y. Harouna-Mayer,[1,2] Francesco Caddeo,[1] Torben Steenbock,[3] Sarah-Alexandra Hussak,[1] Jagadesh Kopula Kesavan,[1] Ann-Christin Dippel,[4] Xiao Sun,[4,5] Andrea Köppen,[6] Viktoriia A. Saveleva,[7] Surender Kumar,[3] Gabriel Bester,[2,3] Pieter Glatzel,[7] and Dorota Koziej[1,2]*

[1] University of Hamburg, Institute for Nanostructure and Solid-State Physics, Center for Hybrid Nanostructures, Luruper Chaussee 149, 22761 Hamburg, Germany

[2] The Hamburg Center for Ultrafast Imaging, 22761 Hamburg, Germany

[3] University of Hamburg, Department of Chemistry, HARBOR, Luruper Chaussee 149, 22761 Hamburg, Germany

[4] Deutsches Elektronen-Synchrotron DESY, Notkestraße 85, 22607 Hamburg, Germany

[5] Institute of Integrated Natural Science, University of Koblenz, Universitätsstraße 1, 56070 Koblenz, Germany

[6] University of Hamburg, Department of Chemistry, Grindelallee 117, 20146 Hamburg, Germany

[7] ESRF, The European Synchrotron, 71 Avenue des Martyrs, CS40220, 38043 Grenoble Cedex 9, France

DFT calculations of vtc-XES and XAS spectra:

The energy positions of the K$\beta_{2,5}$ and white line, as well as the white line intensity in the simulations slightly differ from the experimental data, showing a stronger change than one could observe in the experimental data when comparing the $[Zn(OA)_6]^{2+}$ and $[Zn(SOA)_4]^{2+}$ complexes. This might be caused by inaccuracies in the DFT calculations of those charged complexes. All calculated spectra are redshifted by 18.2 eV for XAS and 14.8 eV for vtc-XES to correct the mismatch between calculations and experimental data.

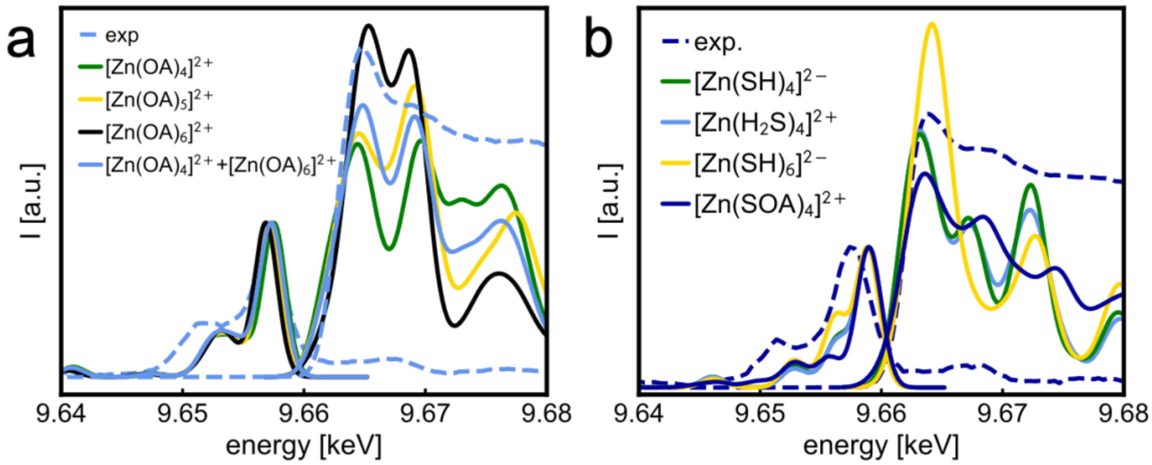

Figure SI1: DFT calculations of different Zn species. Additional calculations of other coordination numbers and chemical environments for **(a)** $[Zn(OA)_4]^{2+}$, $Zn(OA)_5]^{2+}$ and $[Zn(OA)_6]^{2+}$ compared with the mixed tetrahedral $[Zn(OA)_4]^{2+}$ (60%) and octahedral $[Zn(OA)_6]^{2+}$ (40%) complexes and **(b)** $[Zn(SH)_4]^{2-}$, $[Zn(H_2S)_4]^{2+}$, $[Zn(SH)_6]^{2-}$ and $[Zn(SOA)_4]^{2+}$**.** Some complexes such as $[Zn(SOA)_5]^{2+}$, as well as a $[Zn(SOA)_6]^{2+}$ complex, are not compared in this Figure since the molecules explode during geometrical optimization which indicates the instability of those complexes.

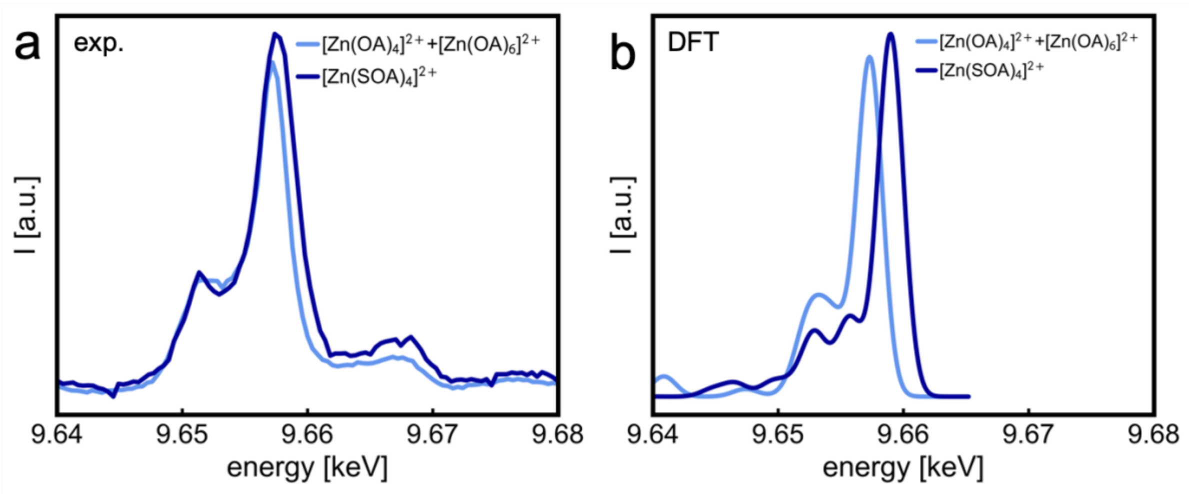

Figure SI2: vtc-XES analysis using area normalization, showing individual transitions and images of the HOMO orbitals. Comparing experimental vtc-XES (**a**) with calculated (**b**) spectra of $[Zn(OA)_4]^{2+}$ and $[Zn(OA)_6]^{2+}$ 60:40 ratio (light blue) and $[Zn(SOA)_4]^{2+}$ (dark blue) complexes, experimental data were normalized by the area. Calculated data were not normalized. The expected trends in intensity and energy position were reproduced in the simulations.

However, this normalization procedure does not work at all for the vtc-XES *in situ* dataset, since short acquisition times decrease the S/N ratio and complicated the baseline fitting, resulting glitches have a higher impact on the total area. Therefore, the intensity of the vtc-XES shows an unphysical intensity difference between individual scans, as shown in **Figure SI24**.

In **Figure SI3**, we show ORCA calculations of a molecular unit of $Zn(Ac)_2$, cut out of a $Zn(Ac)_2$ crystal with the monoclinic phase. The energy positions of the whiteline in the HERFD-XAS and the $K\beta_2$ peak are corroborated. Furthermore, the relative intensity between the $K\beta_5$ and $K\beta_2$ peaks aligns with the experimental data. However, the $K\beta_5$ peak exhibits multiple contributions, where the transition at lower energy (primarily based on dipole transitions from O p => Zn 1s, as shown in **Figure SI5**) is underrepresented in the experimental data. This contributes to the broad nature of the $K\beta_5$ peak, as noted in the first revision comment. A possible reason for overestimating this transition is that, in reality, the oxygen in the third coordination sphere of Zn^{2+} is bonded to another Zn^{2+} ion, which is not accounted for in this ORCA calculation. The lineshape of the edge and post-edge features in the simulated HERFD-XAS spectra does not match the experimental data, which is anticipated when comparing a molecular unit with a macroscopic crystal.

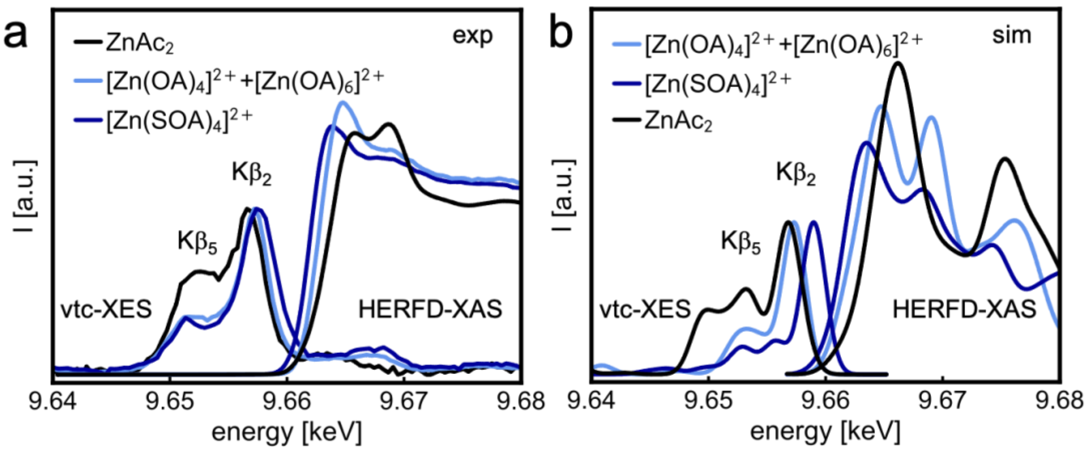

Figure SI3: Experimental (**a**), and simulated (**b**) vtc-XES and HERFD-XAS data of $Zn(Ac)_2$ (black), the $[Zn(OA)_4]^{2+}$ + $[Zn(OA)_6]^{2+}$ (light blue), and $[Zn(OAS)_4]^{2+}$ (dark blue) complexes. The energy shifts of the Kβ2, as well as the relative intensity between the Kβ2 and Kβ5 peaks, are also present in the ORCA calculations. The ORCA calculations reproduce the shift in the whiteline from $Zn(Ac)_2$ to $[Zn(OA)_4]^{2+}$ + $[Zn(OA)_6]^{2+}$, and to $[Zn(OAS)_4]^{2+}$.

To match the simulation with the experimental data, we compared intensities and energy positions of the vtc-XES and HERFD-XAS by different mixing fractions between $[Zn(OA)_4]^{2+}$ and $[Zn(OA)_6]^{2+}$. Those behaviors can be exclusively reproduced by the fractions used 60% $[Zn(OA)_4]^{2+}$ and 40% $[Zn(OA)_6]^{2+}$, which are shown in **Figure SI4**.

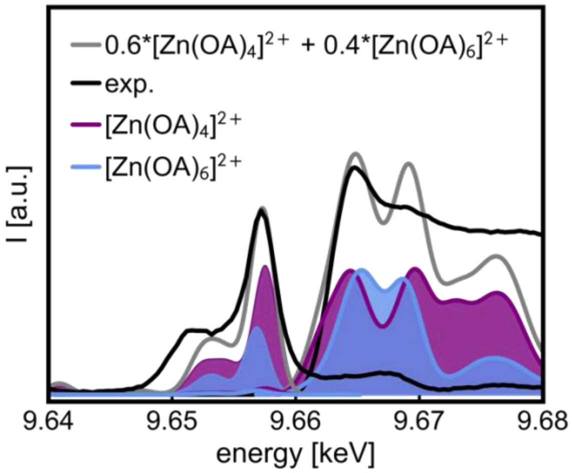

Figure SI4: Comparing experimental data of $Zn(Ac)_2$ in oleylamine with simulated vtc-XES and HERFD-XAS data of a linear combination of 60% $[Zn(OA)_4]^{2+}$ and 40% $[Zn(OA)_6]^{2+}$ (gray), with the individual of contribution of $[Zn(OA)_4]^{2+}$ (purple) and $[Zn(OA)_6]^{2+}$ (blue) to the mixture. The intensity of the vtc-XES and HERFD-XAS simulations were calibrated by comparing simulations of $[Zn(SOA)_4]^{2+}$ with experimental data. The energy position and the

intensity of the Kβ$_2$ peak, as well as the energy position of HERFD-XAS peaks, can be exclusively reproduced by the used fractions of 60% $[Zn(OA)_4]^{2+}$ and 40% $[Zn(OA)_6]^{2+}$.

To evaluate differences between simulated and experimental data, the transitions reflected in the simulated vtc-XES spectra were analyzed in the following **Figure SI5**. Additionally, we visualized the corresponding donor orbitals of the transitions with the strongest contributions.

As shown in **Figure SI5**, by comparing the Kβ$_5$ spectra of $[Zn(SOA)_4]^{2+}$ with those of $[Zn(OA)_4]^{2+}$ and $[Zn(OA)_6]^{2+}$, it becomes apparent that the transition from C p => Zn 1s is not well-represented in the $[Zn(OA)_4]^{2+}$ and $[Zn(OA)_6]^{2+}$ spectra. This might result from the approximation of using a calculated methylamine group instead of the actual oleylamine ligand. The different lengths of the carbon chain might lead to varied donating behaviors of the C p-orbitals. Therefore, the simulated Kβ$_2$ peaks in $[Zn(OA)_4]^{2+}$ and $[Zn(OA)_6]^{2+}$ appear to be narrower than those in the experimental data

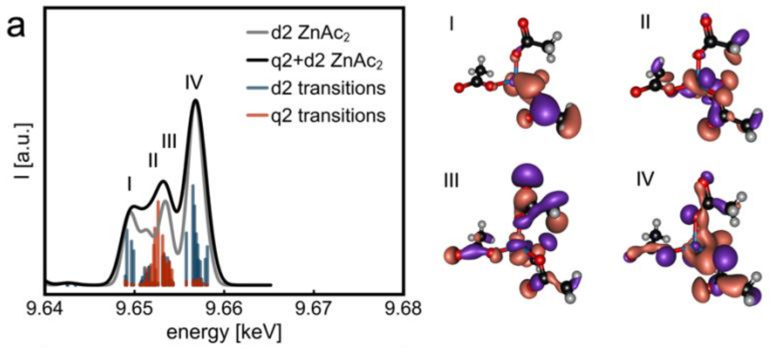

peak	transition	donor orbitals
I	O p => Zn 1s	O px py pz (MO38)
II	Zn d => Zn 1s	Zn dz² dx²y² (MO57)
III	O p => Zn 1s	Zn 6px 6py 6pz O px py pz (MO58)
IV	Zn 6p => Zn 1s	Zn 6px 6py 6pz O px py pz (MO67)

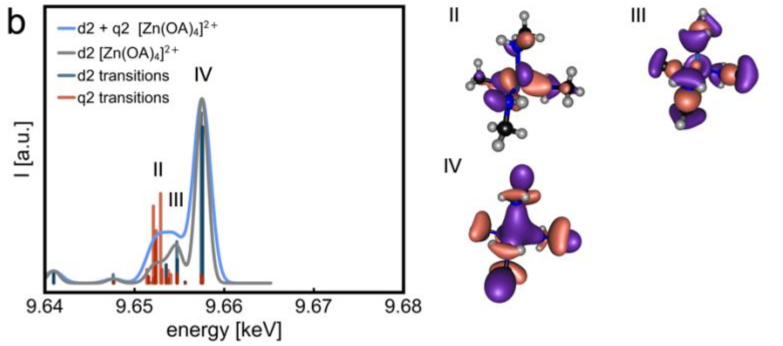

peak	transition	donor orbitals
II	Zn d => Zn 1s	Zn dz² dxy N px py pz (MO31)
III	N p => Zn 1s	Zn dz² dxy N px py pz (MO39)
IV	Zn 6p => Zn 1s	Zn dz² dxy 6px 6py 6pz (MO47)

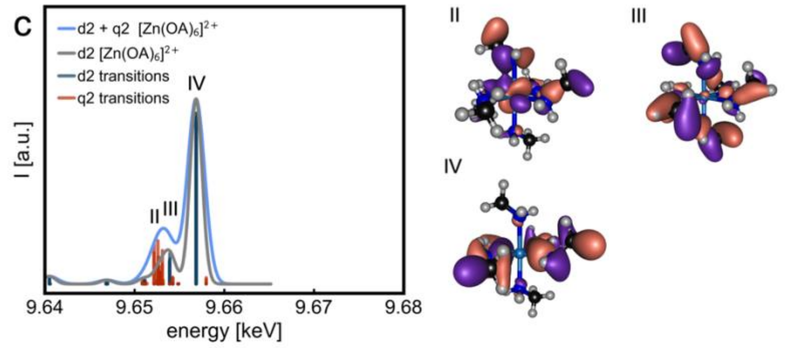

peak	transition	donor orbitals
II	Zn d => Zn 1s	Zn dyz dxy dx²y² N px pz (MO42)
III	N p => Zn 1s	N px py pz (MO52)
IV	Zn 6p => Zn 1s	Zn 6py 6pz N py (MO65)

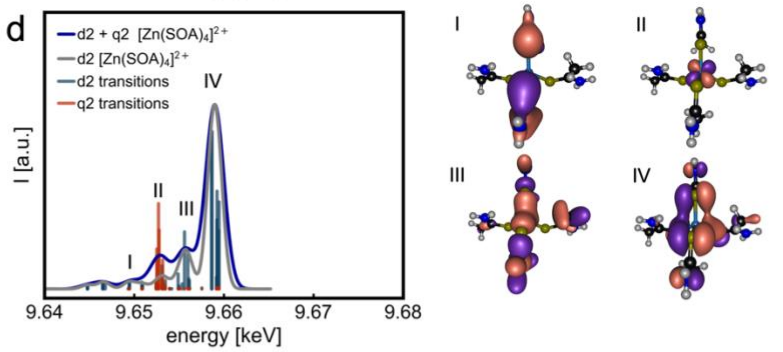

peak	transition	donor orbitals
I	C p => Zn 1s	C px (MO52)
II	Zn d => Zn 1s	Zn dx²y² (MO66)
III	S p => Zn 1s	S px py (MO75)
IV	Zn 6p => Zn 1s	Zn 6py S py pz (MO88)

Figure SI5: (left) Calculated spectra and the dipole and quadrupole contributions for (**a**) $Zn(Ac)_2$, (**b**) $[Zn(OA)_4]^{2+}$, (**c**) $[Zn(OA)_6]^{2+}$, and (**d**) $[Zn(SOA)_4]^{2+}$. The most intense contributions to the vtc-XES spectra are visualized (middle). The transitions responsible for the marked peaks in the vtc-XES spectra are listed in a table (right), along with the donor orbitals of the most intense transitions. A detailed list of all transitions, their intensities, and all corresponding donor orbitals is available in the .out files in the repository, as described earlier and in the SI.

All the structural optimization, as well as the calculations of the vtc-XES (left) and TD-DFT for XAS calculations (right), were performed with the following example ORCA .inp parameters, adapted from *Stepanic et. al*:[1]

```
!RKS B3LYP TightOpt TightSCF ZORA-def2-TZVP ZORA
!FREQ Largeprint Printbasis
%maxcore 4000
%xes
CoreOrb 0
OrbOp 0
end
* xyz 2 1
…
```

```
!RKS B3LYP TightSCF ZORA-def2-TZVP D3BJ RIJCOSX ZORA
!Normalprint MOREAD
%maxcore 4000
%moinp "name.gbw"
%tddft
orbwin[0]= 0,0,-1,-1 doquad true
nroots 300
maxdim 50
end

* xyz 2 1
…
```

A detailed description of the used functionals and parameters can be found in the ORCA input library.[2]

To export the spectra, the resulting output files are processed with orca_mapspc.

XES: orca_mapspc name.out xesq -eV -x09000 -x110000 -n10001 -w2.25

XAS: orca_mapspc name.out absq -eV -x09000 -x110000 -n10001 -kw0.03

The spectra of vtc-XES without quadrupole transitions, shown in Figure SI2, were obtained by:

orca_mapspc name.out xes -eV -x09000 -x110000 -n10001 -w2.25

The .inp Files, the optimized .xyz files of all shown complexes, and the experimental raw data are available under the following DOI: 10.25592/uhhfdm.14737

Coordination of thioamide ligands in Zn[SOA] complexes:

DFT calculations were conducted to identify the coordination of thioamide ligands in the experiment from the two possible coordinations depicted in **Figure SI6**.

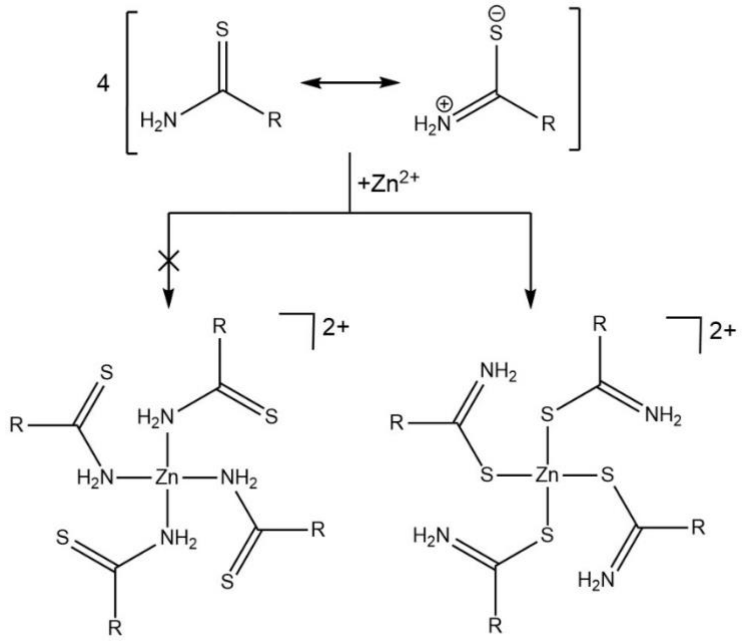

Figure SI6: Illustration of Zn^{2+} coordination in the $[Zn(SOA)_4]^{2+}$ complex. DFT structural optimization reveals that the thioamide group favors the coordination via the sulfur atom.

Thioamides show two resonance structures, one neutral form and one charge-separated form with a negative charge being located on the sulfur atom. The former resonance structure favours the coordination of the thioamide groups via the nitrogen to the Zn(II) cations, while the latter resonance structure favours the coordination via sulfur. In order to see which coordination is preferred, we carried out structure optimizations for both complexes depicted in **Figure SI6.** The optimization of the left and the right structures obtained the sulfur-coordinated complex regardless of the initial structure, which supports that the sulfur-coordinated complex is formed in the experiment, once the oleylamine and sulfur form a thioamide group.

NMR-analysis:

As shown in literature[3], the formation of thioamide in sulfur-amine reaction mixtures is detectable with ^{13}C NMR spectroscopy of reaction mixture aliquots. Analogously to their approach, aliquots from a reaction mixture of sulfur and oleylamine were taken at different temperatures and analyzed by ^{1}H and ^{13}C NMR spectroscopy. The $^{13}C\{^{1}H\}$-NMR spectra are shown in **Figure SI7.**

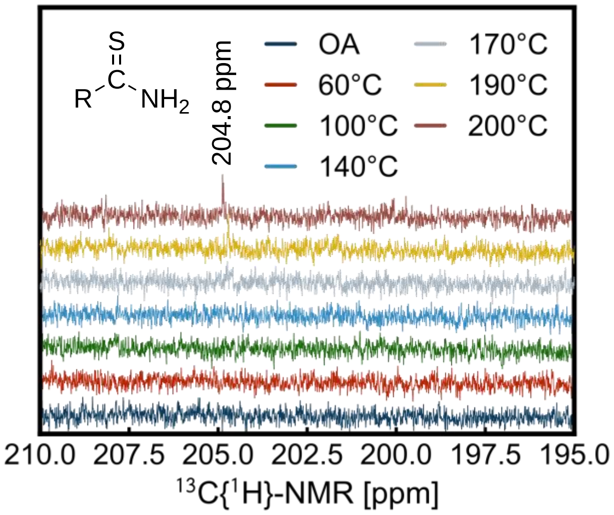

Figure SI7: *ex situ* NMR analysis of aliquots. $^{13}C\{^{1}H\}$-NMR spectra of aliquots taken from a mixture of sulfur (9.1 mmol) in oleylamine (45.5 mmol) indicating the formation of thioamide at temperatures above 170 °C. Spectra of aliquots collected at and below 170 °C and the spectrum of oleylamine in the absence of sulfur do not exhibit a signal corresponding to the thioamide group.

$^{13}C\{^{1}H\}$-NMR spectra of pure oleylamine and reaction mixtures collected at the temperature range of 60-170 °C do not exhibit signals in the downfield from 195—210 ppm. On the other hand, aliquots collected from mixtures heated to 190 °C and 200 °C exhibit a weak signal at 204.8 ppm. Such a peak is an indication of the thioamide formation, since the carbon nucleus of a thioamide functional group is expected to occur in the downfield of around 204 ppm.[3]

The aliquot collected from the reaction mixture of $Zn(Ac)_2$, sulfur and oleylamine at room temperature was measured with an increased number of scans (NS=10240) to improve the signal-to-noise ratio, which increases as the square root of the number of scans. Despite an increased number of scans, the $^{13}C\{^{1}H\}$-NMR spectrum (**Figure SI8**) does not exhibit a signal in the range of 200—210 ppm, which could correspond to a thioamide.

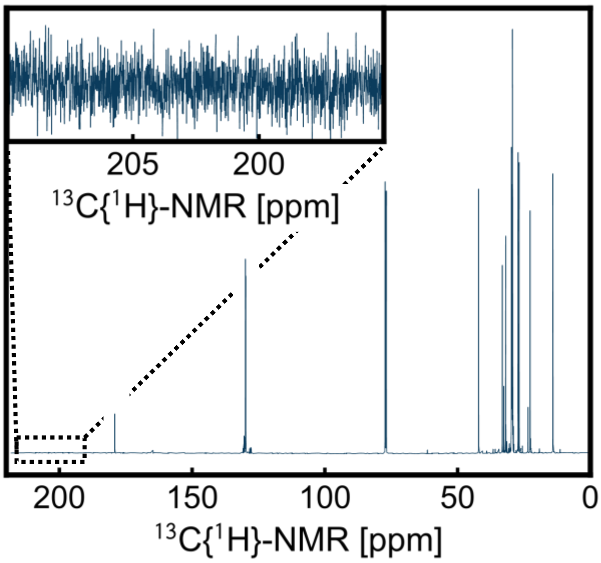

Figure SI8: *ex situ* NMR analysis at room temperature. $^{13}C\{^{1}H\}$-NMR spectrum acquired with 10240 scans of the reaction mixture $Zn(Ac)_2$ (6.65 mmol), S (9.1 mmol) and oleylamine (45.4 mmol) at room temperature. The spectrum does not exhibit a peak above 200 ppm which could indicate the formation of a thioamide functional group.

MCR-ALS analysis:

The multivariate curve resolution-alternating least squares (MCR-ALS) method employs a constrained alternating least squares (ALS) algorithm to solve the bilinear model. The bilinear equation is expressed as follows:

$$\mathbf{D} = \mathbf{C}\mathbf{S}^{\mathbf{T}} + \mathbf{E} \quad (1)$$

where **D** represents the raw data set, **C** denotes the concentration profiles for each recovered compound, $\mathbf{S}^{\mathbf{T}}$ stands for the related pure spectra, and **E** is the matrix of residuals not accounted for by the model. The ALS algorithm operates on the principle of alternately fixing one set of variables while solving for the other. Specifically, the process begins by initializing $\mathbf{S}^{\mathbf{T}}$ and solving a linear algebra problem for **C** while keeping $\mathbf{S}^{\mathbf{T}}$ constant. Subsequently, **C** is held constant, and the system is solved for $\mathbf{S}^{\mathbf{T}}$. This cycle is repeated until convergence is achieved. Convergence is defined by the condition that, in two consecutive iterative cycles, the relative differences in the standard deviation of residuals between experimental and ALS-calculated data values are less than a pre-determined threshold. Quality assessments of a converged fit are derived from the unexplained residuals **E**, representing the disparity between the experimental data and the bilinear model, with the **lack of fit (%)** expressed as:

$$\textbf{lack of fit (\%)} = 100\sqrt{\frac{\sum_{i,j} e_{ij}^2}{\sum_{i,j} d_{ij}^2}} \quad (2)$$

where **d** denotes an element of the data matrix, and e corresponds to the respective element in the residuals matrix **E**. Furthermore, the variance $\mathbf{R^2}$ explained in the model can be estimated using:

$$\mathbf{R}^2 = \frac{\sum_{i,j} d_{ij}^2 - \sum_{i,j} e_{ij}^2}{\sum_{i,j} d_{ij}^2} \quad (3)$$

The standard deviation of the residuals $\boldsymbol{\sigma}$ is given by:

$$\boldsymbol{\sigma} = \sqrt{\frac{\sum_{i,j} e_{ij}^2}{nm}} \quad (4)$$

Here, *n* and *m* denote the dimensions of the raw data matrix **D**.

Furthermore, the ALS algorithm can adhere to physically or chemically meaningful constraints, such as non-negativity or unimodality for concentration and spectra profiles, as well as closure.[4] To conduct the MCR-ALS analysis, the XAS data were organized into a matrix, where each row corresponds to an absorption spectrum recorded *in situ*. The number of rows equals the quantity of acquired spectra, and the number of columns corresponds to the number of data points in each spectrum. Prior to implementing the ALS optimization, the determination of the number of components and initial spectra was undertaken. The selection of the number of components was based on the outcomes of Singular Value Decomposition (SVD). The computed eigenvalues from the data provide insights into the extent of variance each factor or component can elucidate. The factor associated with the highest eigenvalue exhibits the greatest variance, while the one with the second highest eigenvalue follows, and so forth. Eigenvalues below a certain threshold are indicative of noise in the data. The determined Eigenvalues in both shown datasets are plotted as Scree plot in **Figure SI9** and are listed in **Table SI2**.

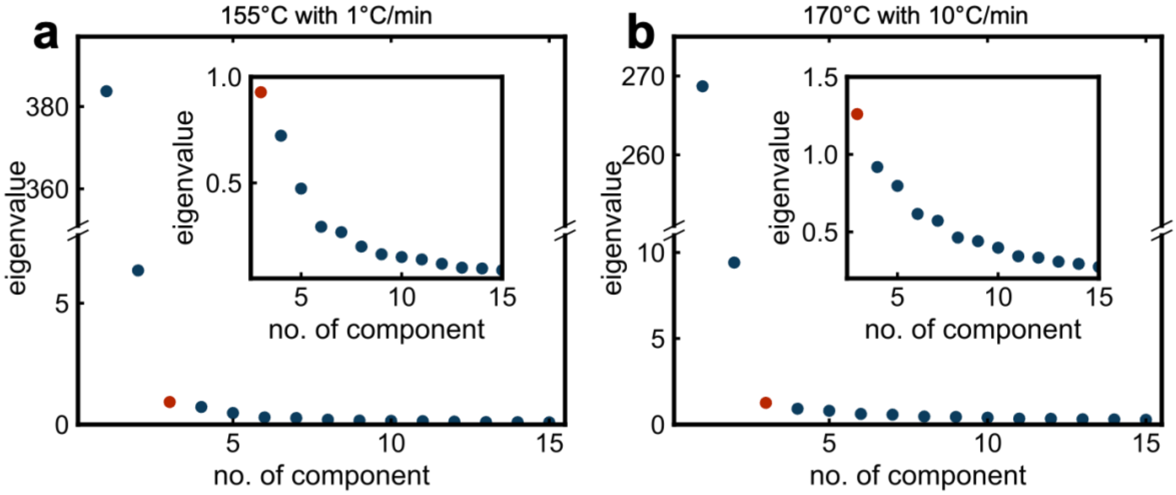

Figure SI9: Eigenvalues of MCR-ALS analysis. Scree plot of eigenvalues of the ZnS reaction with reaction temperature of **(a)** 155 °C with heating rate of 1 °C/min and of **(b)** 170 °C with a heating rate of 10 °C/min. The alignment of eigenvalues of four or more components that belong to noise. The red dot marks the last used component.

Table SI1: Eigenvalues determined by SVD of the MCR-ALS analysis.

Number of components	Eigenvalues of synthesis at 155 °C – 1°C/min (Fig. SI6a)	Eigenvalues of synthesis at 170 °C - 10°C/min (Fig. SI6b)
**1**	383.717	268.701
**2**	6.351	9.415
**3**	0.928	1.260
**4**	0.723	0.918
**5**	0.474	0.797
**6**	0.294	0.616

Table SI2: Fitting parameters of the MCR-ALS analysis.

	Synthesis at 155 °C – 1°C/min (Fig. 2b)	Synthesis at 170 °C - 10°C/min (Fig. SI17)
**fitting error (%)**	2.505	2.360
**R^2**	99.937	99.944
**σ**	0.023	0.022

Owing to the limited solubility of $Zn(Ac)_2$ in oleylamine, the initial 22 spectra at room temperature have been excluded from the data matrix D for the experiment conducted at a reaction temperature of 155 °C and a ramping rate of 1°C/min. This exclusion is necessary because fluctuations in Zn concentrations introduce non-physical features. To facilitate the recovery process during the MCR-ALS analysis of ZnS nanocluster formation, the spectrum of ZnS nanoparticles was appended as the final spectrum.

During the MCR-ALS analysis, two to four different components were checked, and the results were investigated in detail. To discriminate meaningful analysis, we followed this selection procedure:

1. Setting the convergence criteria: We set criteria for a meaningful MCR-ALS analysis, e.g., that the concentration of individual components cannot be negative, and show unimodal behavior. Also, the addition of all concentrations at each timestep of the reaction has to be 100%. Further, the XANES spectra cannot run negative.

a. The MCR-ALS analysis with four components does not achieve convergence with these criteria and can be excluded.

2. Of every MCR-ALS analysis that achieved a convergence (here, for two and three components) the spectral profile was checked if unphysical features might appear. (e.g. pre-edges in Zn K-Edge)
   a. For two and three components, the resulting spectral profiles did not show any unphysical behavior
3. If multiple MCR-ALS analyses achieve convergence and show physically meaningful spectral profiles, the quality of reproducing the first spectra of the reaction with the first component can give rise to the total recovering quality.
   a. The two-component MCR-ALS shows a high fitting error and cannot explain all spectral changes. As shown in the following **Figure SI10**, the starting point was recovered with two components in a crude way.

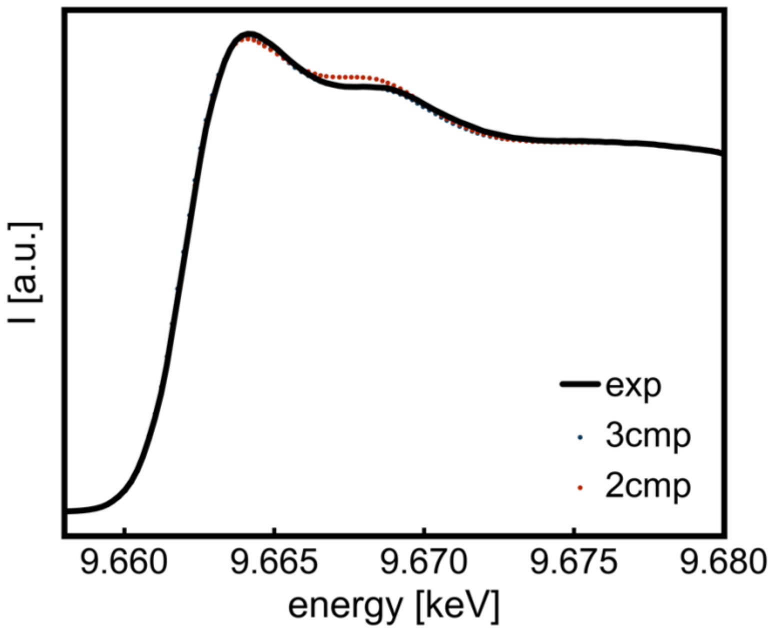

Figure SI10. The first component of MCR-ALS analysis uses in total three components (blue) and two components (red). The experimental starting complex (black) was reproduced with low error by three-component analysis, while the two-component analysis crudely recovered the first experimental spectra.

Band gap analysis of HERFD-XAS/vtc-XES data:

The resonance energy is material-dependent and changes during the reaction, thus for the *in situ* studies in solution, we excite non-resonant at 9.7 keV and collect the vtc-XES spectra. We are comparing the ionized HOMO and the non-ionized LUMO to calculate the energy difference indicated in red in **Figure 2c**. Thus, the E_{gap} is systematically underestimated by about 1.5 eV. The difference between the minimum of the derivative of the XES data and the maximum of the derivative of the HERFD-XAS data reflects the HOMO/LUMO gap at every reaction time.

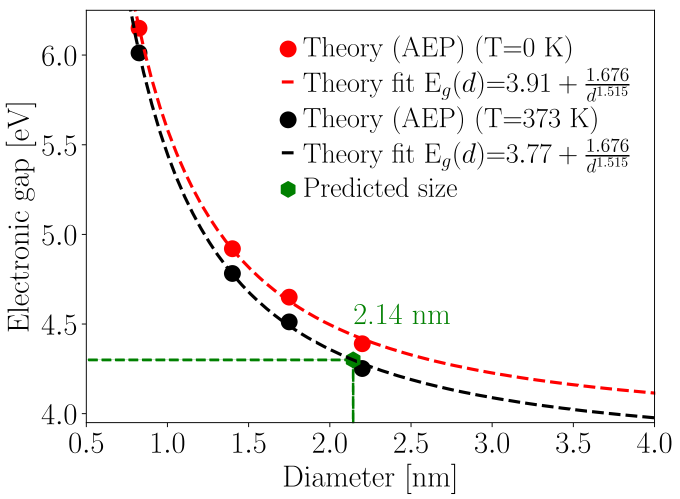

Figure SI11: Electronic band gap of w-ZnS as a function of diameter. Electronic band gap of wurtzite ZnS quantum dots (QDs) as a function of diameter, calculated using atomic effective pseudopotentials (AEPs) (see text for details). The filled red (black) circles are the electronic gaps at T=0 K (372 K) and the red (black) solid line is a diameter-dependent fit. The green dot shows our measured electronic gap and the ensuing dot diameter.

We calculate the single-particle (SP) electron and hole eigenenergies and wavefunctions using atomic effective pseudopotentials (AEPs) including spin-orbit coupling effects.[5, 6] The AEPs are derived from density functional theory (DFT) calculations and subsequently corrected for the electronic band gap.[7] The quantum dots (QDs) are passivated with fractional charge non-spherical pseudo-hydrogen atoms.[8] This approach has often been used successfully in the past to calculate optical and electronic gaps.[7, 9, 10]

Since our SP calculations are performed at zero temperature, we include temperature effects *a posteriori* via an analytical expression for the temperature dependence of the experimental band gap presented in Literature:[11]

The parameters used in the equation are given as:

$$E(T) = E(0) - \frac{\alpha\Theta_\rho}{2}\left\{\frac{\rho}{2}\left(\left(\sqrt[4]{1+\frac{\pi^2}{6}\left(\frac{4T}{\rho}\right)^2+\left(\frac{4T}{\rho}\right)^4}\right)-1\right)+(1-\rho)\left[coth\left(\frac{\Theta_\rho}{2T}\right)-1\right]\right\}$$

E(0) = 3.91 eV

α = 0.548 meV/K

Θ_ρ = 350 K

ρ = 0.389

This approach leads to a 138 meV red shift of the band gap when going from zero to room temperature (373 K). This value has been subtracted from our calculated band gap. With this procedure, we obtain a QD diameter of 2.1 nm corresponding to our measured electronic gap of 4.3 eV, which is consistent with our dot size distribution.

UV-VIS analysis:

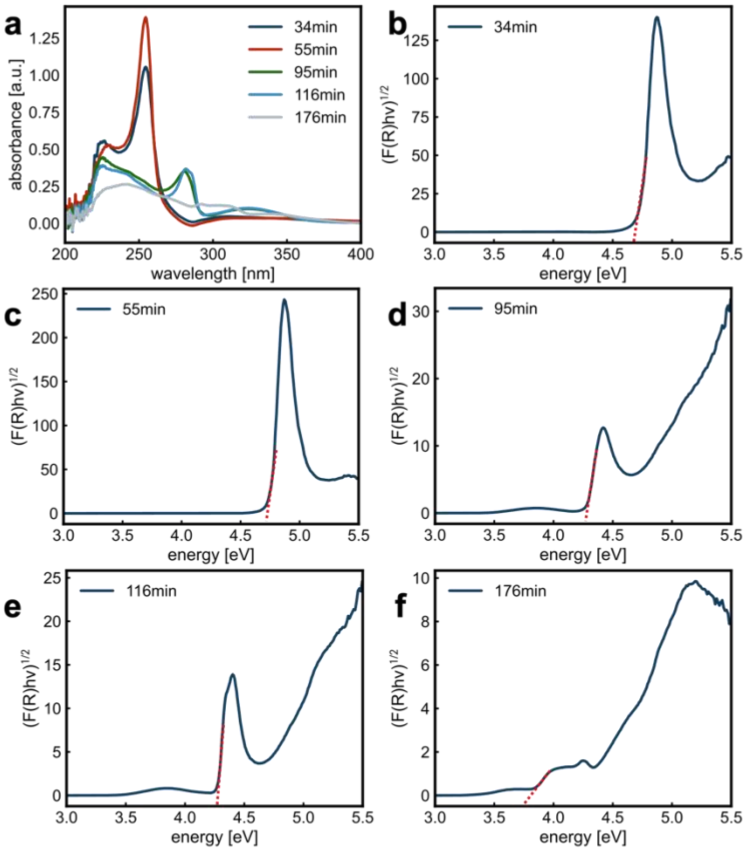

Figure SI12: *ex situ* UV-VIS analysis. (a) UV-Vis spectra of the quenched reaction at various time steps. The reaction times of 34, 55, 95, 116, and 176 min correspond to the reaction temperatures of 75, 95, 135, 155 and 155 °C, respectively. **(b-f)** Tauc-Plot analysis from the UV-vis data, providing the values for the optical band gap, summarized in **Table SI3** and shown in **Figure 2**. Since the synthesis of s-ZnS undergoes the co-preparation of w-ZnS as well as possible organic byproducts, one can observe multiple peaks in the range of 3.8 eV to 4.5 eV, especially at 116min and 176 min.

The linear fit has the general equation of y = ax+b. The values for the intercept and slope, and the calculated band gap for each reaction step, are summarized in **Table SI3**.

Table SI3: Values of fitting the Tauc-plots from UV-Vis data and the corresponding band gap.

	**34 min - 75 °C**	**55 min - 95 °C**	**95 min - 135 °C**	**116 min - 155 °C**	**176 min - 155 °C**
**a**	-1170.4 ± 123	-1841 ± 392	-413.18 ± 22.7	-638.38 ± 55	-24.84 ± 0.51
**b**	251.21 ± 26.2	392.49 ± 47.3	96.685 ± 5.27	149.51 ± 12.8	6.54 ± 0.13
**ΔE**	4.66 eV	4.69 eV	4.28 eV	4.27 eV	3.79 eV

SAXS background processing:

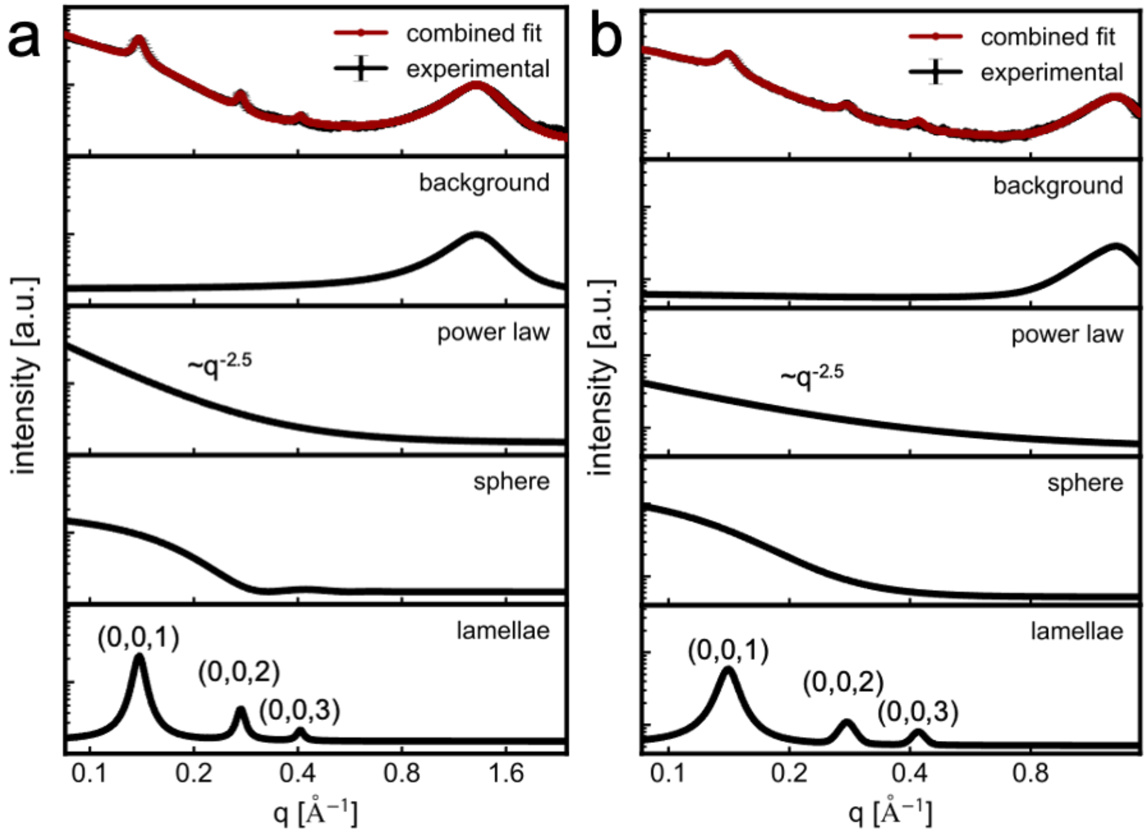

Figure SI13: SAXS background subtraction in the synthesis at (a) 155°C with 1°C/min ramping rate and (b) 170°C and 10°C/min ramping rate. SAXS fitting of a representative scan (top), exhibiting the individual contributions (background, power law that is treated as part of the background, sphere to represent the NP and the lamellae formed by the solvent) The lamellae induce a sharp peak with 2-5 multiplets which is observed in the SAXS signal. This behavior is well known from lipid bilayers and has been reported for thio-derivatives of oleylamine, with a bilayer distance around 38 Å.[12] Nevertheless, the lamellae occur only during the synthesis of ZnS NC, as shown in **Figure SI14**.

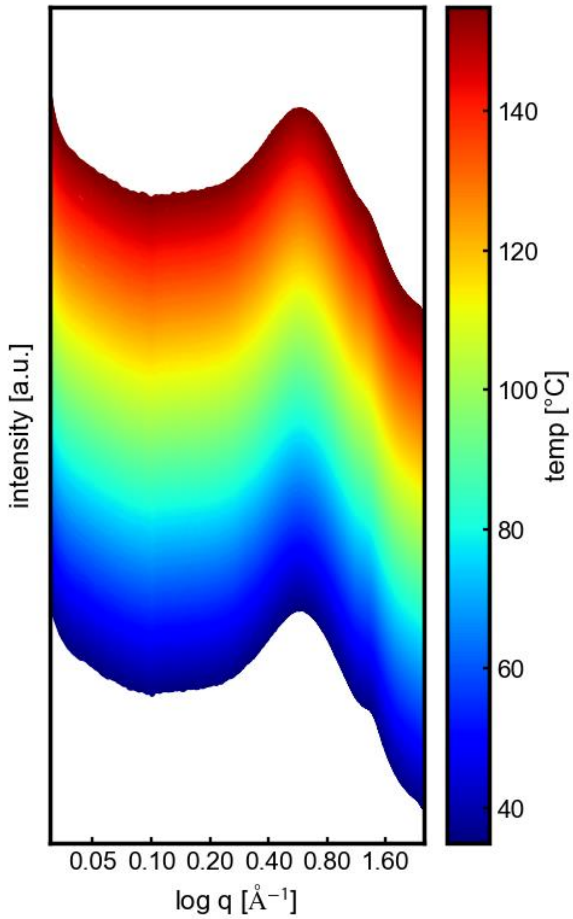

Figure SI14: Control *in situ* SAXS data of oleylamine and sulfur solution heated to 155°C with 1°C/min ramping rate. Unlike in **Figure 3a** where $Zn(ac)_2$ is present in solution, the lamellas do not form.

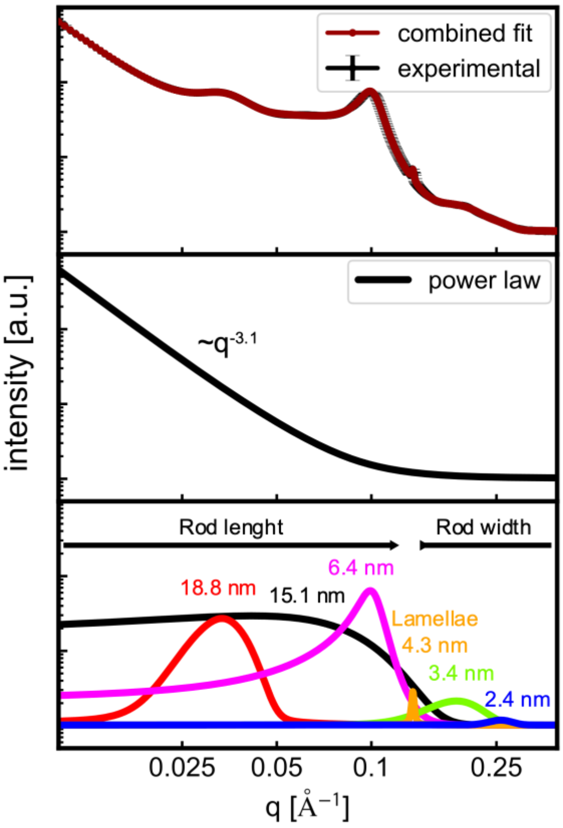

Figure SI15: SAXS fit of the reaction mixture after cooling down. Measured with a lower q-min to visualize the limitations of the used *in situ* SAXS dataset. (top) deconvoluted into the power law (middle) and individual size distributions (bottom) of the ZnS NP. All larger sizes with contributions lower than 0.08 $Å^{-1}$ are not accessible by *in situ* SAXS. The high energy is mandatory for achieving a high q_{max} in the SAXS/WAXS combined detector setup at P07.[13] The combined SAXS/WAXS signal is shown in **Figure SI16**.

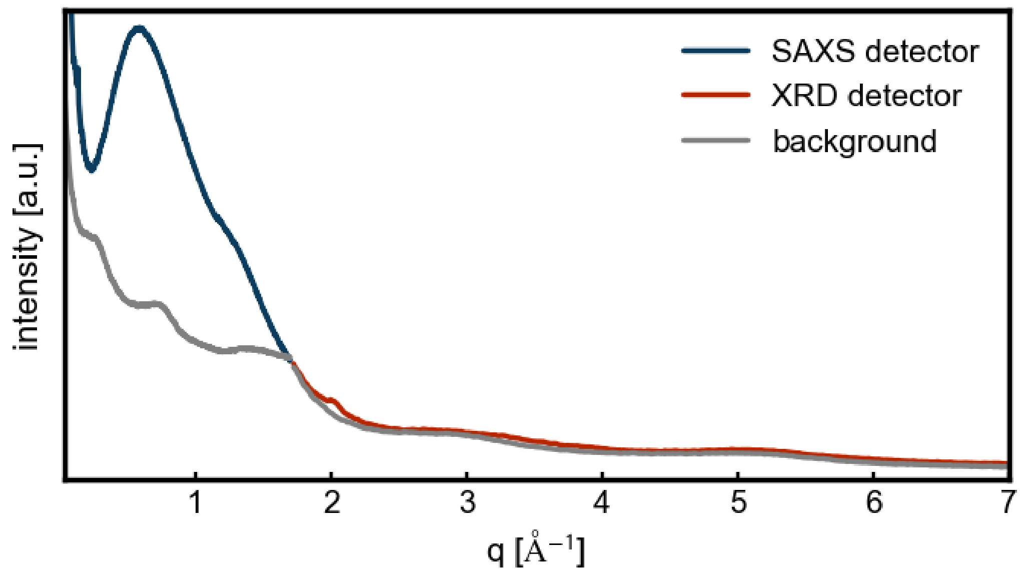

Figure SI16: Simultaneous acquired SAXS and PXRD data. Simultaneous acquired SAXS (blue) and PXRD (red) data of the reaction solution after 250 min of the reaction, with the background of oleylamine and sulfur (gray) at 155°C.

***Ex situ* characterization of s-ZnS:**

The size of the s-ZnS nanorods is described by STEM analysis as 3.1 ± 1.4 nm width and 10.8 ± 2.8 nm length, while the domain size, calculated by Rietveld analysis of PXRD data, reveals a domain size of 3nm x 6nm, as shown in **Figure SI17**. Consequently, the nanorods mostly consist of multiple crystalline domains, attached along the s(111) axis.

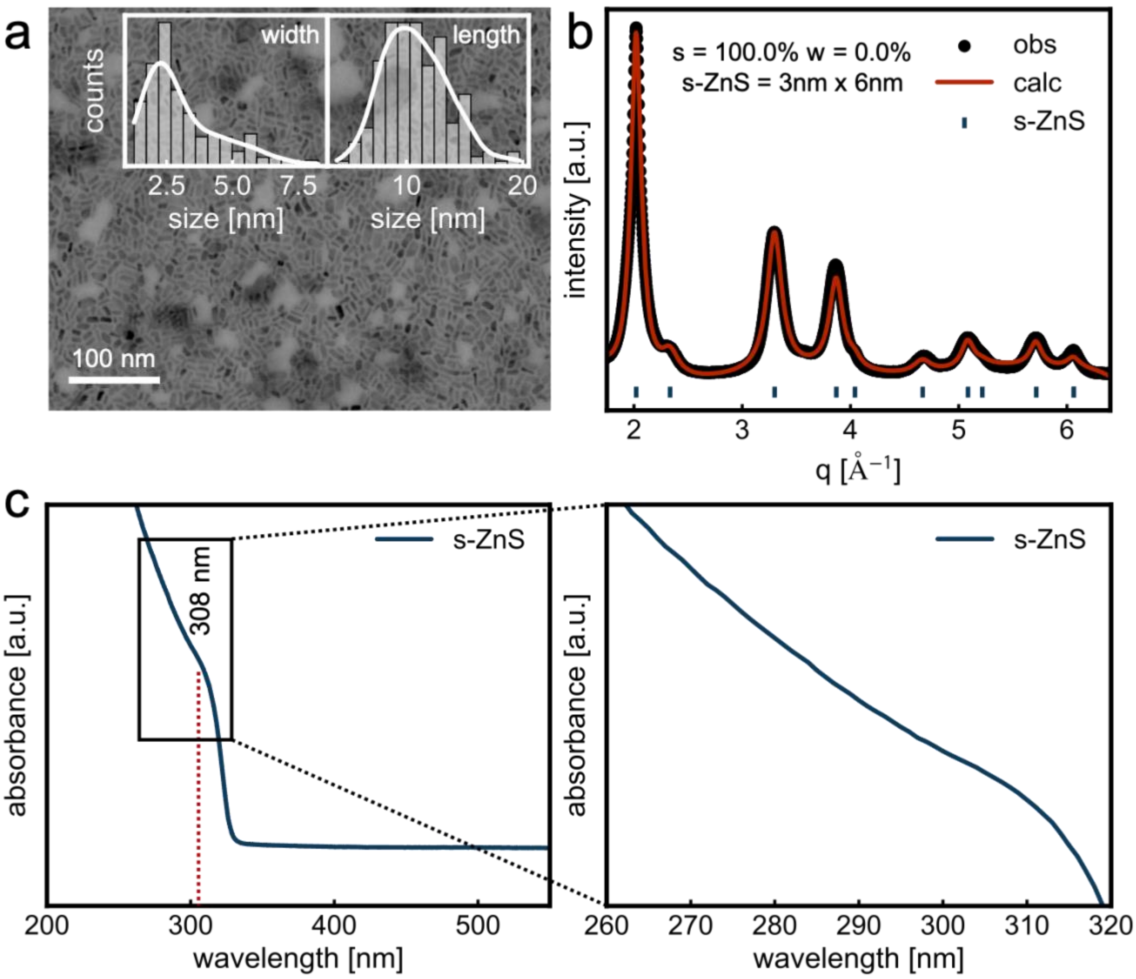

Figure SI17: STEM image, Rietveld refinement and UV-Vis analysis of the final product after washing procedure. (a) STEM analysis shows nanorods with a size of 3.1 ± 1.4 nm width and 10.8 ± 2.8 nm length. In the inset, we present a size histogram based on the analysis of 207 particles. **(b)** Rietveld analysis of PXRD data of the final product conclude phase pure ZnS nanorods in sphalerite phase with domain sizes of 3nm x 6nm. (c) UV-VIS analysis shows a rod-like absorption behavior with a peak of s-ZnS at 308nm.[14]

Upon the initiation of sphalerite ZnS formation, rod-like structures in the sphalerite phase are observed, with dimensions approximately 2 nm in width and 7 nm in length. By the end of the reaction, the s-ZnS grows to a thickness of 3.1 ± 1.4 nm and a length of 10.8 ± 2.8

nm, as described in **Figure SI17**. To enhance the image quality of the s-ZnS shown in **Figure SI18 and Figure 3b (top, middle)**, the aliquots were washed before the deposition on a TEM grid.

HRTEM analysis of the s-ZnS:

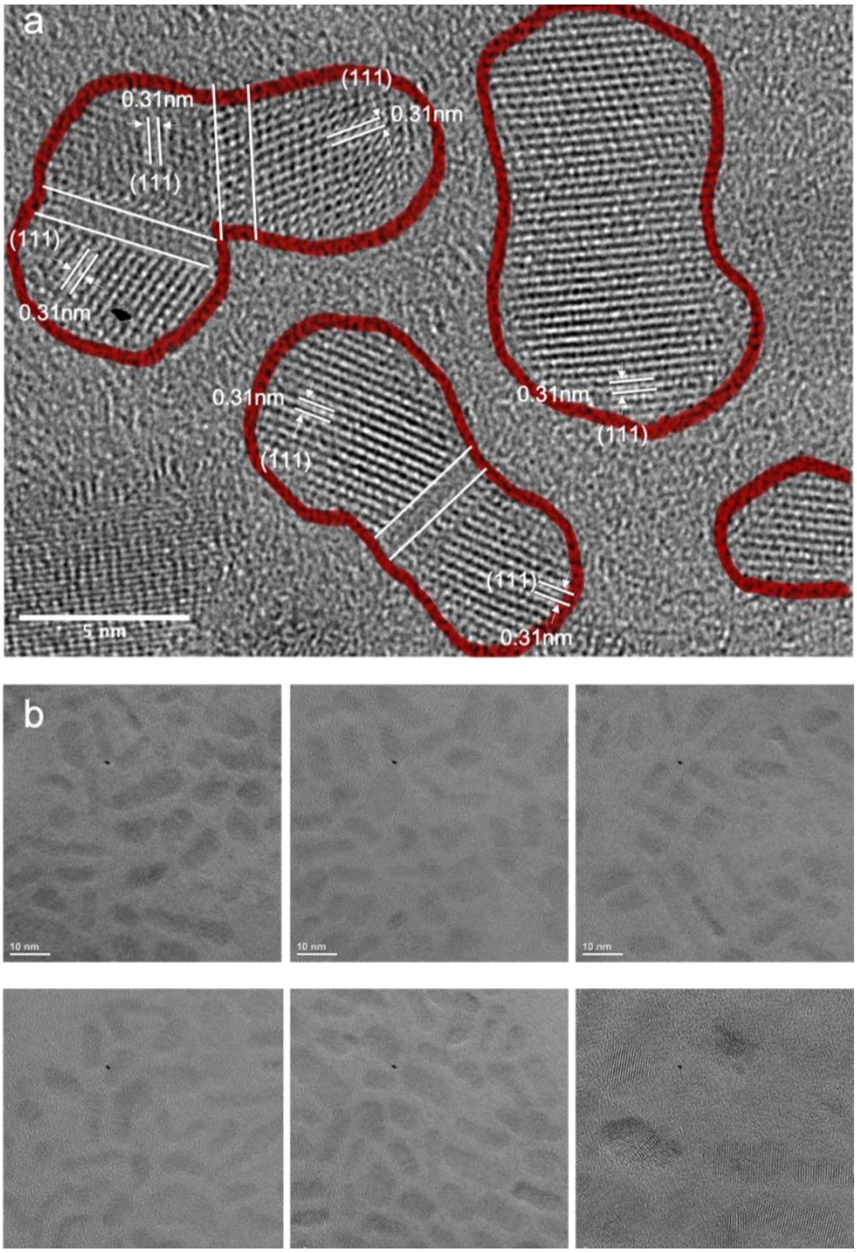

Figure SI18: HRTEM analysis of the s-ZnS. HRTEM images of an aliquot at 155 min after eliminating the w-ZnS contribution by washing procedures. (a) The lattice fringes are indexed to the ZnS sphalerite phase. Contour lines highlighting the shape of the s-ZnS. (b) Overview of HRTEM pictures at different spots on the grid. The analysis reveals that the elongated structures seen in the HRTEM images are the s-ZnS. We assume that w-ZnS nanoparticles agglomerate/assemble before transforming into s-ZnS rods. The lattice fringes suggest an intergrowth process rather than dissolution-recrystallization.

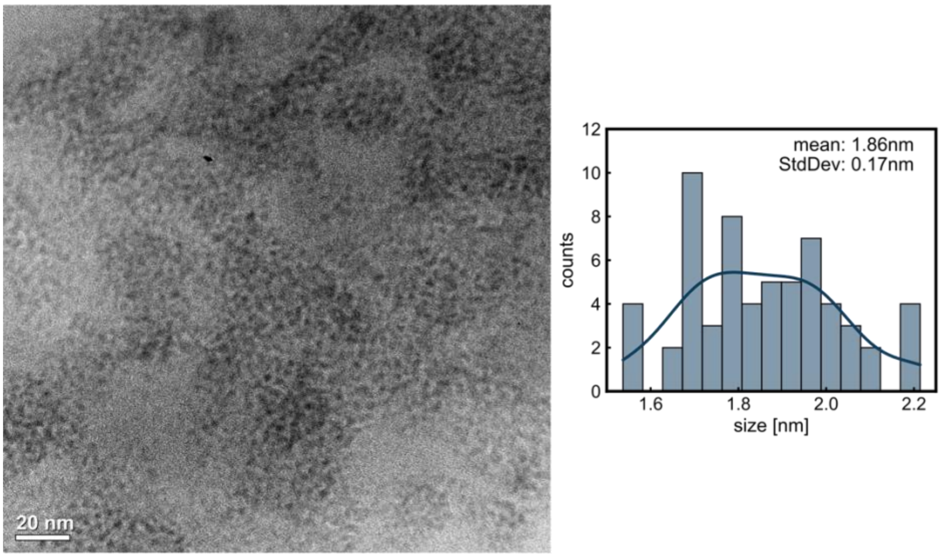

Figure SI19: HRTEM analysis of the w-ZnS. HRTEM images of an unwashed aliquot at 75min reaction temperature, reveal 1.9 ± 0.2 nm large particles, which belong to w-ZnS.

Sequential Rietveld refinement:

Rietveld refinement utilizes the peak widths of reflections in the diffraction pattern to determine the crystallite sizes of simulated reference patterns through least-squares analysis. In this study, the diffraction pattern of the final reaction product could not be adequately described by a single ZnS phase. Consequently, we implemented a two-phase refinement, incorporating a linear combination of two simulated diffraction patterns corresponding to wurtzite ZnS (w-ZnS) and sphalerite ZnS (s-ZnS). We observed discrepancies in peak intensities compared to simulations, notably the (111) reflection of s-ZnS appeared narrower and exhibited higher intensity relative to other reflections in the pattern. To address this, we modeled anisotropy in s-ZnS along the (111) orientation. The least-squares fitting process optimized the proportions of w-ZnS and s-ZnS, as well as their respective crystallite sizes.

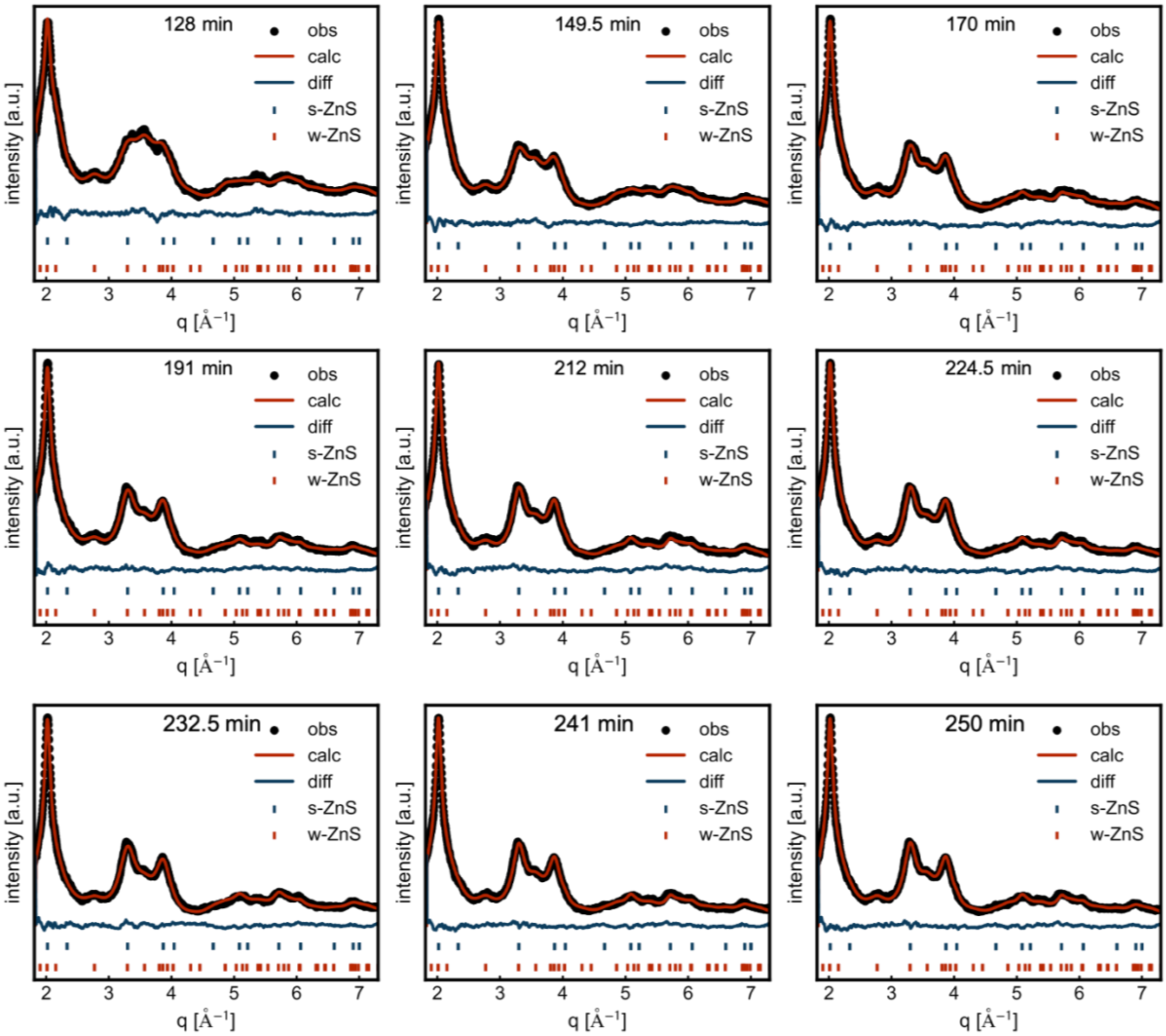

Figure SI20: Sequential Rietveld refinement of PXRD. The data analysis assigns changes in the reflexes during the reaction to changes in domain sizes and fractions between s-ZnS and w-ZnS, as summarized in **Table SI4**.

Table SI4: Rietveld refinement results at different times of the reaction.

	Rw	s-ZnS	w-ZnS	size s-ZnS	size w-ZnS
**128 min**	4.55 %	15.7%	84.3 %	1 nm x 7 nm	1.7 nm
**149.5 min**	3.62 %	29.3%	70.7 %	1 nm x 7 nm	1.9 nm
**170 min**	4.77 %	32.3 %	67.7 %	2 nm x 7 nm	1.8 nm
**191 min**	4.04 %	33.9 %	66.1 %	1 nm x 7 nm	1.9 nm
**212 min**	5.69 %	36.4 %	63.6 %	2 nm x 7 nm	1.9 nm
**224.5 min**	4.35 %	38.8 %	61.2 %	2 nm x 7 nm	1.8 nm
**232.5 min**	5.53 %	38.6 %	61.4 %	1 nm x 7 nm	1.9 nm
**241 min**	4.46 %	39.9 %	60.1 %	2 nm x 7 nm	1.9 nm
**250 min**	4.33 %	41.0 %	59.0 %	2 nm x 7 nm	2.0 nm

In situ vtc-XES, HERFD-XAS, SAXS and PXRD characterization of the synthesis of s-ZnS at 170°C with a heating rate of 10°C/min:

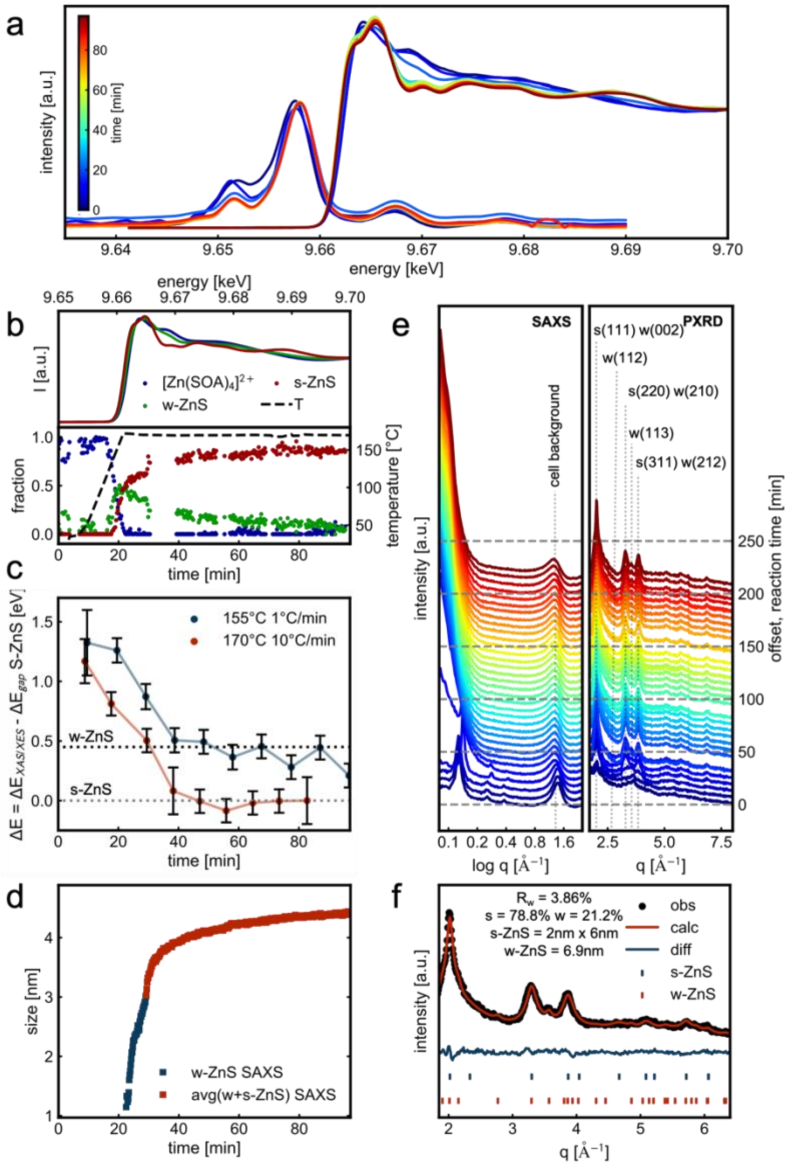

Figure SI21: _In situ_ X-ray spectroscopy, SAXS and PXRD analysis of synthesis at 170°C with 10°C/min heating rate.

(a) *in situ* vtc-XES and HERFD XAS, analyzed by MCR-ALS in **(b)**. The recovered spectra matched with the MCR-ALS of the in **Figure 3b**. The *in situ* band gap calculation in **(c)** suggests a faster formation of s-ZnS which is in good agreement with the MCR-ALS data. **(d)** The size was calculated by fitting the SAXS data with a spherical model. **(e left)** *In situ* SAXS analysis. The increasing intensity at low q, starting after approximately 20 minutes, indicates the formation of nanoparticles during the synthesis. **(e right)** *In situ* PXRD analysis shows the formation of crystalline reflections after 20 minutes, with changes in the relative intensity of the w(210)/s(220) and w(113) peaks over time, suggesting the formation of the sphalerite phase

during the reaction. The s(111) peak appears to be comparably intense, implying a preferred orientation in the s along the (111) facet. **(f)** Rietveld analysis of the final product shows the presence of 79% s-ZnS and 21% w-ZnS.

***In situ* reaction cell:**

To run the reactions at the beamlines, adapted versions of reaction cells from previous work were used.[15, 16] **Figure SI22a** presents the body of the *in situ* cell. For *in situ* HERFD-XAS and vtc-XES studies, a PEEK reaction container (diameter 2.5mm) surrounded by a flat, thin wall (0.2 mm) for X-ray entrance and exit was used to obtain enough signal from diluted solutions but with enough mechanical stability (see **Figure SI22a**). For the combined X-ray scattering measurements, an adapted PEEK inlet works as a holder for a glass capillary, described in detail in the literature.[16] The glass capillaries have a 0.5 mm wall thickness and 2.5 mm inner diameter. A detailed view of the inlet used in the X-ray scattering experiments is shown in **Figure SI22b**.

The total volume of 66 µL and 174 µL for the scattering and spectroscopic experiments, respectively. The main potential differences can occur in different heat capacities and therefore different heat ratios. To estimate the impact on the total heat consumption of the cell, we calculated the required energy to heat 66 µL and 174 µL of oleylamine (in the assumption that oleylamine has a comparable specific heat capacity to oleic acid of 2.4 $\frac{\mathrm{kJ}}{\mathrm{kg*K}}$) to 155°C to 16.1 J and 42.5 J respectively. Concerning the required energy of around 23 kJ to heat our heating cell to 155°C, the impact of the sample volume is below 0.2%. Therefore, we did not expect any impact of the sample volume on our reaction kinetics.

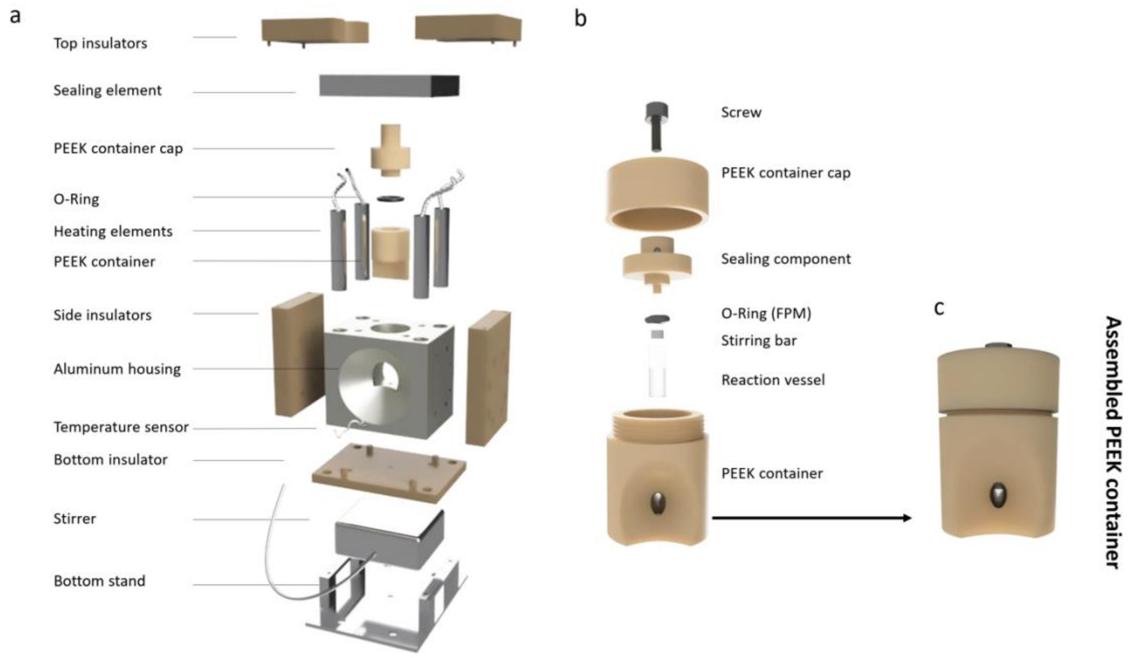

Figure SI22: Schematic of the used reaction container. (a) Unassembled view of the reaction cell, used in X-ray spectroscopy experiments. **(b)** Adapted PEEK inlet with glass capillary as a reaction vessel, used in X-ray scattering experiments.

Beam damage study:

To exclude the occurrence of beam damage, we performed radiation damage studies before the measurement, collecting multiple XANES spectra at the same position on the pellet/cell and checking if any changes in the spectra due to the radiation can be observed.

$Zn(Ac)_2$ powder diluted in boron nitride and pressed to pellet.

We observed rapid radiation damage during XANES scans, as shown in **Figure SI23a**. The occurrence of radiation damage led us to limit the acquisition time of an XANES to 6s, and average the XANES over 40 XANES spectra from different points on the pellet.

Zn solution: In the XANES spectra of $Zn(Ac)_2$ dissolved in oleylamine, we do not see any effect of radiation damage, as shown in the **Figure SI23b**. We collected 20 XANES spectra on the same spot in the *in situ* cell, where every XANES scan took 10s. To mitigate the beam damage during the reaction: we moved the *in situ* cell between the scans to an exposed spot in the reaction container and stirred during the reaction.

vtc-XES studies: For each datapoint in the vtc-XES scan a fresh spot on the pellet was used with a collection time of 1s per spot. To perform a concentration correction on the vtc-XES dataset, we moved again to all measured spots and collected the intensity of the Kβ main. The intensity variations on the vtc-XES are based on inhomogeneities in the pellet, or thickness of

the in-situ cell. The thickness inhomogeneity in the cell relates to the precision of the machining of the *PEEK* container.

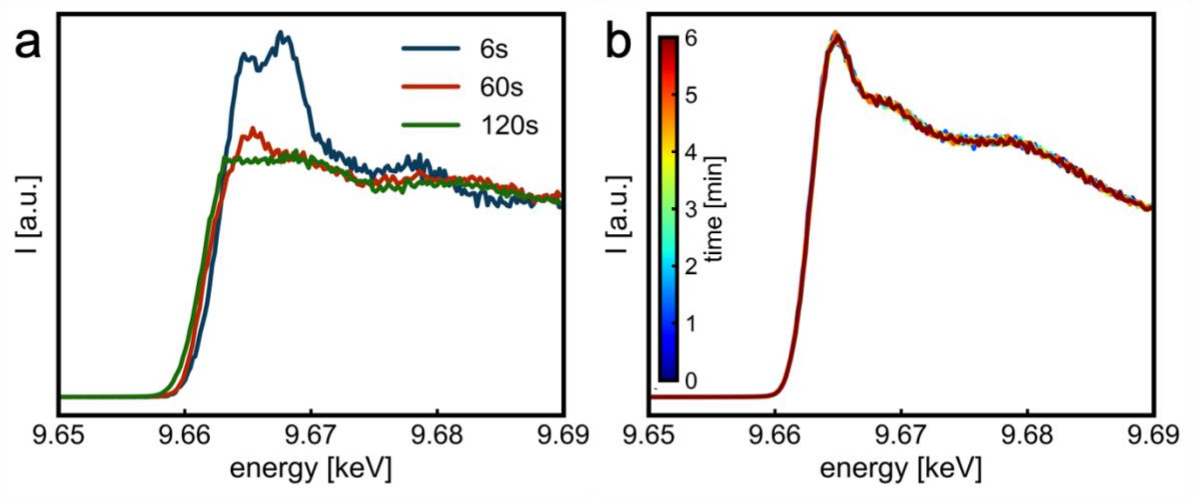

Figure SI23. Radiation damage study on $Zn(Ac)_2$ pellet (a) and $Zn(Ac)_2$ dissolved in oleylamine (b). While pure $Zn(Ac)_2$ shows a fast occurrence of beam damage, the $Zn(Ac)_2$ dissolved in oleylamine seems unaffected by incident X-ray radiation.

Normalization of vtc-XES:

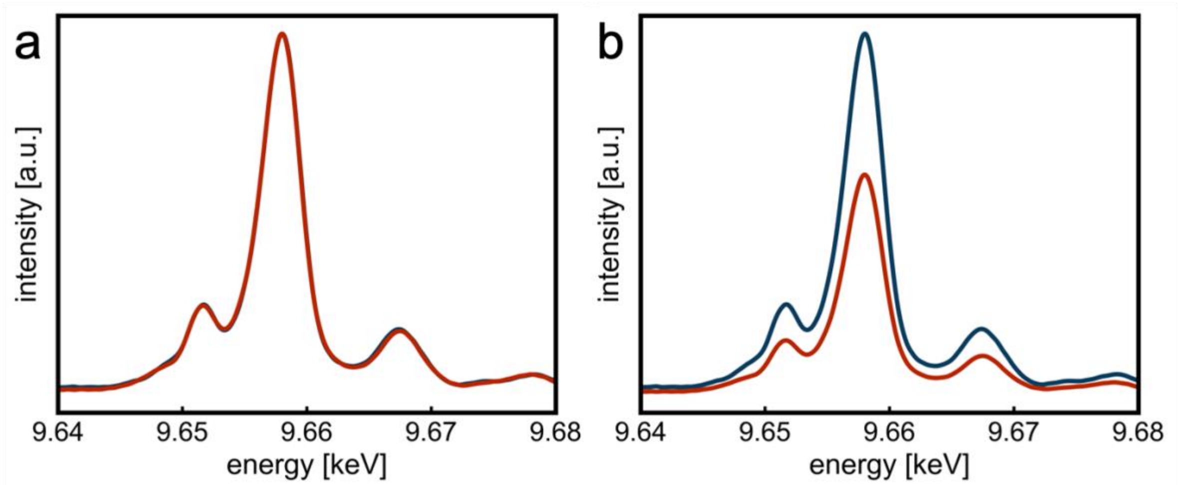

Figure SI24: *In situ* vtc-XES after smoothing and interpolation (as explained in Figure SI22a) with normalization by max intensity (a), show two almost identical scans at the end of the reaction, while an area normalization (b) would show an unphysical overall intensity difference.

In summary, the most elegant way to normalize the *in situ* data was a normalization of the intensity of the Kβ main line. But this would lead to a significant decrease in time resolution.

Thus, we maintained normalization by maximum intensity rather than by area, which allows us to compare the relative intensities between the individual peaks and avoid unphysical misinterpretation of intensities due to glitches.

FEFF calculations of vtc-XES and data processing:

We perform FEFF calculations[17] of w-ZnS and s-ZnS to check if we would expect large changes between the vtc-XES spectra. The calculations reveal only small changes in maximum intensity as shown in the following **Figure SI25**. In General, the limitation of FEFF to reproduce experimental spectra is limited, as described in the literature.[18]

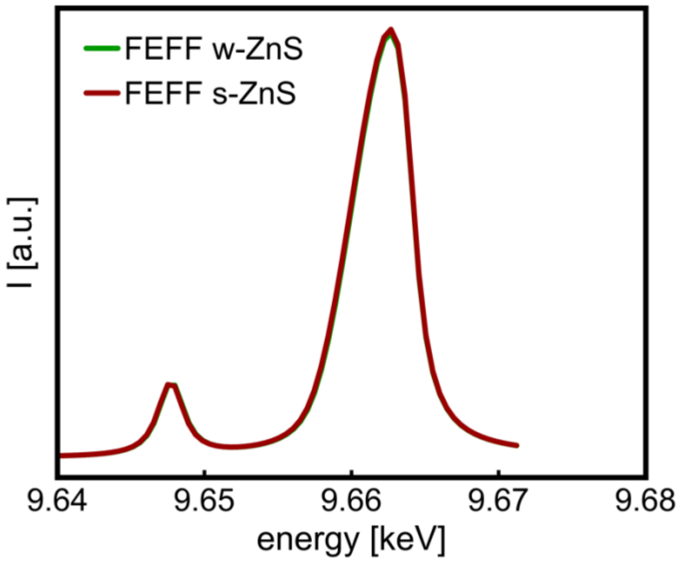

Figure SI25. FEFF simulation of w-ZnS and s-ZnS reveals only small changes in maximum intensity.

The FEFF calculation were performed with the following parameters:

```
EDGE K
S02 1.0
CONTROL 1 1 1 1 1 1
PRINT 1 0 0 0 0 3
XES 4.0 0.005 0.0
EXCHANGE 0 0.0 0.0 -1
SCF 9 0 100 0.2 10
COREHOLE FSR
LDOS -20 30 0.5
MULTIPOLE 2 0
```

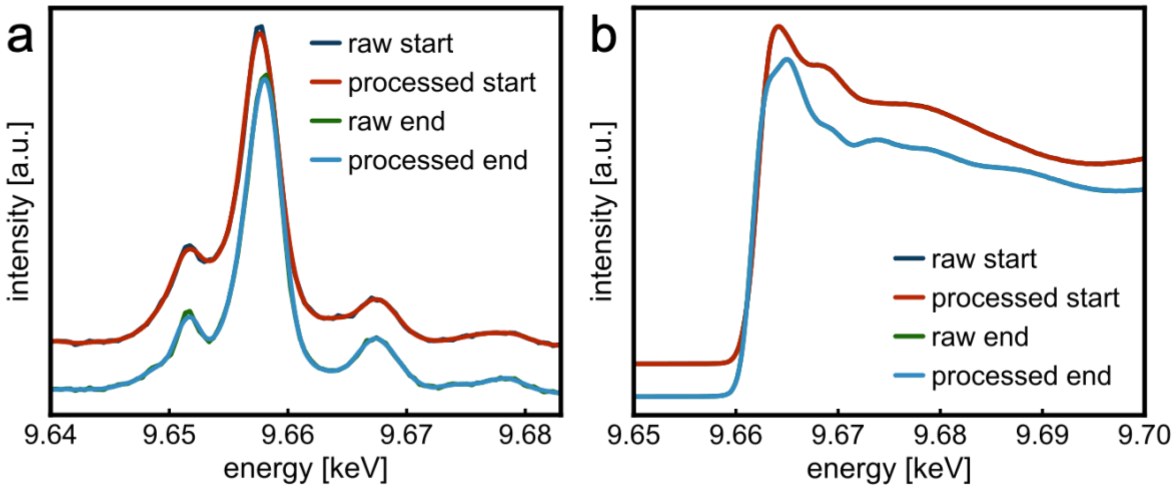

Figure SI26: Data processing in HERFD-XAS and vtc-XES data. vtc-XES **(a)** and HERFD-XAS data **(b)** were interpolated and filtered as described in the experimental section. This data optimization was required to ensure a reliable determination of the energy position of the maximum in the derivation. To verify that the data were not significantly affected by the processing, the filtered and raw data at the beginning and the end of the *in situ* runs are combined and plotted with an offset.

References:

(1) McCubbin Stepanic, O.; Ward, J.; Penner-Hahn, J. E.; Deb, A.; Bergmann, U.; DeBeer, S. Probing a Silent Metal: A Combined X-ray Absorption and Emission Spectroscopic Study of Biologically Relevant Zinc Complexes. *Inorganic Chemistry* **2020**, *59* (18), 13551-13560. DOI: 10.1021/acs.inorgchem.0c01931.

(2) Bjornsson, R. *ORCA Inout Library*. 2013. https://sites.google.com/site/orcainputlibrary/home?authuser=0 (accessed 2024 16.04.).

(3) Thomson, J. W.; Nagashima, K.; Macdonald, P. M.; Ozin, G. A. From Sulfur−Amine Solutions to Metal Sulfide Nanocrystals: Peering into the Oleylamine−Sulfur Black Box. *Journal of the American Chemical Society* **2011**, *133* (13), 5036-5041. DOI: 10.1021/ja1109997.

(4) Jaumot, J.; Gargallo, R.; de Juan, A.; Tauler, R. A graphical user-friendly interface for MCR-ALS: a new tool for multivariate curve resolution in MATLAB. *Chemometrics and Intelligent Laboratory Systems* **2005**, *76* (1), 101-110. DOI: 10.1016/j.chemolab.2004.12.007.

(5) Cárdenas, J. R.; Bester, G. Atomic effective pseudopotentials for semiconductors. *Physical Review B* **2012**, *86* (11), 115332. DOI: 10.1103/PhysRevB.86.115332.

(6) Zirkelbach, F.; Prodhomme, P. Y.; Han, P.; Cherian, R.; Bester, G. Large-scale atomic effective pseudopotential program including an efficient spin-orbit coupling treatment in real space. *Physical Review B* **2015**, *91* (7), 075119. DOI: 10.1103/PhysRevB.91.075119.

(7) Kumar, S.; Bui, H.; Bester, G. Empirical band-gap correction for LDA-derived atomic effective pseudopotentials. *Computational Condensed Matter* **2024**, *40*, e00917. DOI: 10.1016/j.cocom.2024.e00917.

(8) Karpulevich, A.; Bui, H.; Antonov, D.; Han, P.; Bester, G. Nonspherical atomic effective pseudopotentials for surface passivation. *Physical Review B* **2016**, *94* (20), 205417. DOI: 10.1103/PhysRevB.94.205417.

(9) Bester, G. Electronic excitations in nanostructures: an empirical pseudopotential based approach. *Journal of Physics: Condensed Matter* **2009**, *21* (2), 023202. DOI: 10.1088/0953-8984/21/2/023202.

(10) Karpulevich, A.; Bui, H.; Wang, Z.; Hapke, S.; Palencia Ramírez, C.; Weller, H.; Bester, G. Dielectric response function for colloidal semiconductor quantum dots. *The Journal of Chemical Physics* **2019**, *151* (22). DOI: 10.1063/1.5128334 (acccessed 7/24/2024).

(11) Pässler, R.; Griebl, E.; Riepl, H.; Lautner, G.; Bauer, S.; Preis, H.; Gebhardt, W.; Buda, B.; As, D. J.; Schikora, D.; et al. Temperature dependence of exciton peak energies in ZnS, ZnSe, and ZnTe epitaxial films. *Journal of Applied Physics* **1999**, *86* (8), 4403-4411. DOI: 10.1063/1.371378 (acccessed 9/17/2024).

(12) Tzitzios, V.; Dimos, K.; Lelidis, I.; Boukos, N. K.; Wadi, V. S.; Basina, G.; Nounesis, G.; Alhassan, S. M. Sulfur–oleyl amine platelet derivatives with liquid crystalline behavior. *RSC Advances* **2018**, *8* (72), 41480-41483, 10.1039/C8RA08325H. DOI: 10.1039/C8RA08325H.

(13) Derelli, D.; Caddeo, F.; Frank, K.; Krötzsch, K.; Ewerhardt, P.; Krüger, M.; Medicus, S.; Klemeyer, L.; Skiba, M.; Ruhmlieb, C.; et al. Photodegradation of CuBi2O4 Films Evidenced by Fast Formation of Metallic Bi using Operando Surface-sensitive X-ray Scattering. *Angewandte Chemie International Edition* **2023**, *62* (43), e202307948. DOI: 10.1002/anie.202307948.

(14) Dai, L.; Lesyuk, R.; Karpulevich, A.; Torche, A.; Bester, G.; Klinke, C. From Wurtzite Nanoplatelets to Zinc Blende Nanorods: Simultaneous Control of Shape and Phase in Ultrathin ZnS Nanocrystals. *The Journal of Physical Chemistry Letters* **2019**, *10* (14), 3828-3835. DOI: 10.1021/acs.jpclett.9b01466.

(15) Staniuk, M.; Hirsch, O.; Kränzlin, N.; Böhlen, R.; van Beek, W.; Abdala, P. M.; Koziej, D. Puzzling Mechanism behind a Simple Synthesis of Cobalt and Cobalt Oxide Nanoparticles: In Situ Synchrotron X-ray Absorption and Diffraction Studies. *Chemistry of Materials* **2014**, *26* (6), 2086-2094. DOI: 10.1021/cm500090r.
(16) Derelli, D.; Frank, K.; Grote, L.; Mancini, F.; Dippel, A.-C.; Gutowski, O.; Nickel, B.; Koziej, D. Direct Synthesis of CuPd Icosahedra Supercrystals Studied by In Situ X-Ray Scattering. *Small n/a* (n/a), 2311714. DOI: 10.1002/smll.202311714.
(17) Rehr, J. J.; Kas, J. J.; Vila, F. D.; Prange, M. P.; Jorissen, K. Parameter-free calculations of X-ray spectra with FEFF9. *Physical Chemistry Chemical Physics* **2010**, *12* (21), 5503-5513, 10.1039/B926434E. DOI: 10.1039/B926434E.
(18) Mortensen, D. R.; Seidler, G. T.; Kas, J. J.; Govind, N.; Schwartz, C. P.; Pemmaraju, S.; Prendergast, D. G. Benchmark results and theoretical treatments for valence-to-core x-ray emission spectroscopy in transition metal compounds. *Physical Review B* **2017**, *96* (12), 125136. DOI: 10.1103/PhysRevB.96.125136.
